# Identification of Genes that Control Silk Yield by RNA Sequencing Analysis of Silkworm (*Bombyx mori*) Strains of Variable Silk Yield

**DOI:** 10.3390/ijms19123718

**Published:** 2018-11-22

**Authors:** Yue Luan, Weidong Zuo, Chunlin Li, Rui Gao, Hao Zhang, Xiaoling Tong, Minjin Han, Hai Hu, Cheng Lu, Fangyin Dai

**Affiliations:** State Key Laboratory of Silkworm Genome Biology, Key Laboratory of Sericultural Biology and Genetic Breeding, Ministry of Agriculture, College of Biotechnology, Southwest University, Chongqing 400715, China; luanyue987@163.com (Y.L.); hnzwd2004@126.com (W.Z.); lclin13@163.com (C.L.); 17347669621@163.com (R.G.); zhang2296690949@163.com (H.Z.); xltong@swu.edu.cn (X.T.); minjinhan@126.com (M.H.); huhaiswu@163.com (H.H.); lucheng@swu.edu.cn (C.L.)

**Keywords:** silk yield, RNA-seq, multiple strains, PCA

## Abstract

Silk is an important natural fiber of high economic value, and thus genetic study of the silkworm is a major area of research. Transcriptome analysis can provide guidance for genetic studies of silk yield traits. In this study, we performed a transcriptome comparison using multiple silkworms with different silk yields. A total of 22 common differentially expressed genes (DEGs) were identified in multiple strains and were mainly involved in metabolic pathways. Among these, seven significant common DEGs were verified by quantitative reverse transcription polymerase chain reaction, and the results coincided with the findings generated by RNA sequencing. Association analysis showed that BGIBMGA003330 and BGIBMGA005780 are significantly associated with cocoon shell weight and encode uridine nucleosidase and small heat shock protein, respectively. Functional annotation of these genes suggest that these play a role in silkworm silk gland development or silk protein synthesis. In addition, we performed principal component analysis (PCA) in combination with wild silkworm analysis, which indicates that modern breeding has a stronger selection effect on silk yield traits than domestication, and imply that silkworm breeding induces aggregation of genes related to silk yield.

## 1. Introduction

Domestic silkworm (*Bombyx mori*) is an economically important insect that was domesticated more than 5000 years ago. In China, the total direct income of silkworm farmers was 111.54 billion yuan in the past five years, the output value of enterprises above a designated size was 641.3 billion yuan, and the export value of real silk products was 16.6 billion US dollars [1]. In addition, silk fibroin has recently been used as a biomaterial and biomedicine for the development and utilization of bone tissue engineering, silk fibroin hydrogels, implant coatings, and nanomaterials [2,3,4,5,6]. The huge industrial value and broad application prospects of silk has thus prompted researchers to develop techniques to improve its yield. Today, traditional breeding methods have been unable to further improve silk quantity. It is thus imperative to use advanced molecular biology methods to identify genes that control silk yield.

Silk traits are quantitative traits that are regulated by multiple genes, and their genetic mechanisms are complex. Currently, quantitative trait loci (QTLs) for multiple cocoon quality traits have been mapped, laying the foundation for gene detection [7,8,9,10,11,12]. Our group previously developed a suitable localization method for the special genetic characteristics of silkworm and detected five loci or chromosomes that were related to cocoon shell weight (CSW) [13]. In addition to constructing QTL maps, several genes related to cocoon quality traits have also been identified using biological methods, such as genome sequencing. Li et al. detected a gene that was related to silk yield by combining a sequencing-based methodology and association analysis [14]. Although these investigations have largely promoted the study of cocoon quantity traits, no silk yield-related genes have been identified and functionally characterized to date.

The silk gland of silkworm, an organ that produces silk, is composed of the anterior silk gland, the middle silk gland (MG), and the posterior silk gland (PG). The middle silk gland and the posterior silk gland produce sericin and silk fibroin, respectively, and silk sericin encapsulates silk fibroin to form silk fibers. The development of the silk gland is closely related to silk yield. Using the silk gland as a material, the researchers screened genes related to silk gland development through transcriptomics and proteomics analyses. Li et al. performed transcriptome analysis using two strains with significant differences in silk yield, namely, Jingsong and Lan10, and detected 1375 DEGs related to silk yield. GO and KEGG analyses indicated that most of these genes are related to protein synthesis [15]. Domestic and wild silkworms also exhibit huge differences in silk yield. Fang et al. compared the transcriptome data of domestic and wild silkworms and found that genes related to protein secretion, metabolism, and tissue development were upregulated during domestication [16]. In addition, silk yield at the protein and micro-RNA levels have been investigated [17,18]. However, due to the fact that these studies use a single pair of strains and that genetic background largely differs among strains, it is possible that some of the screened genes are related to background differences. Therefore, it is essential to use multiple strains with significant differences in cocoon quantity traits for transcriptome analysis to avoid background differences.

We performed RNA sequencing (RNA-seq) using three high-yield and three low-yield strains. Comparisons were made between high- and low-yield strains to obtain nine groups of DEGs, and the number of DEGs varied from 8 to 261. GO and KEGG analyses were performed. To identify more relevant DEGs, we further considered the differential expression of genes between two high-yield strains and two low-yield strains as common DEGs (common DEGs). In addition, we performed Principal Component Analysis (PCA) using wild silkworm data from other investigations and data from this study. The results imply that silkworm breeding induces the aggregation of genes related to silk yield.

## 2. Results

### 2.1. Phenotypic Assessment of Cocoons of Various Silkworm Strains

**Figure 1:**
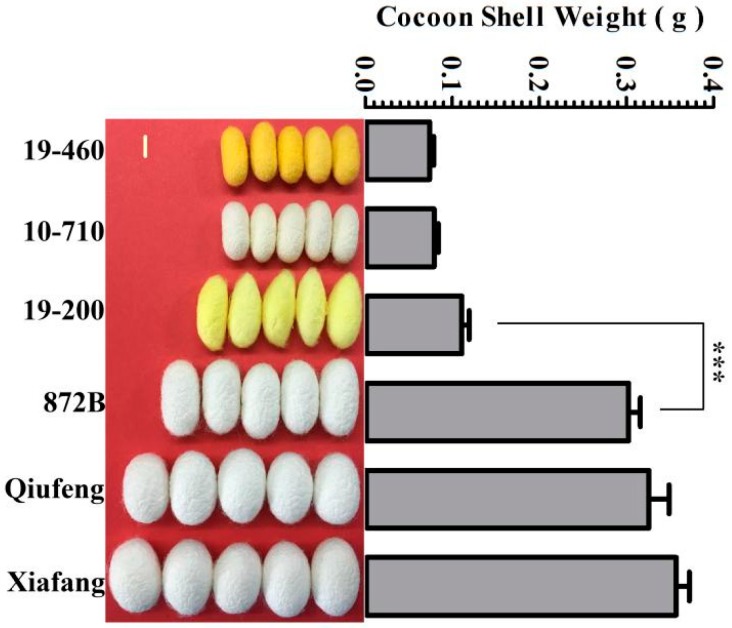
Phenotyping survey of silkworm cocoons. 19-460, 10-710, and 19-200 are low-yield strains of the silkworm. 872B, Qiufeng, and Xiafang are high-yield strains of the silkworm. *t*-Test between high-yield and low-yield strains, ***: *p* ≤ 0.001.

**Figure 2:**
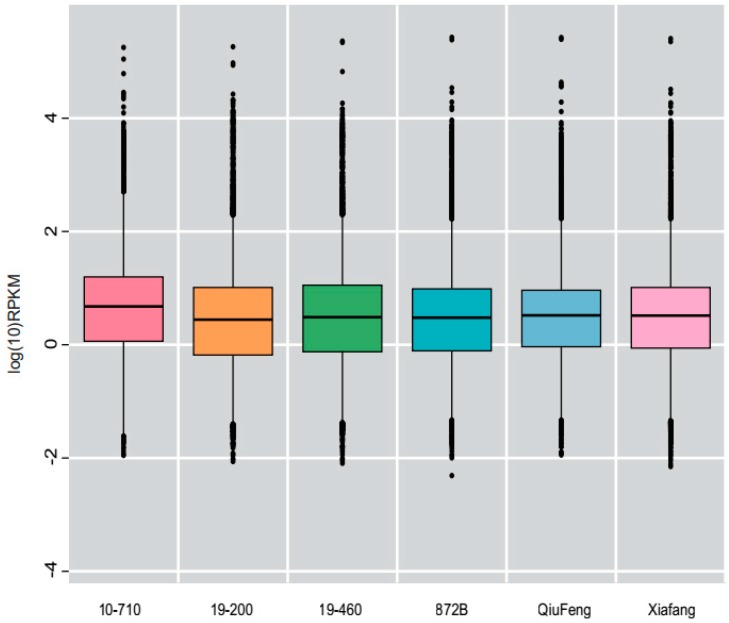
Boxplot of the log transformed RPKM expression values of six silkworm strains. RPKM: Reads per kilobases per million reads. The solid horizontal line represents the median, and the box encompasses the lower and upper quartiles.

This study used three high-yield strains (872B, Qiufeng, and Xiafang) and three low-yield strains (19-200, 10-710, and 19-460) with significantly different cocoon quantity traits (Figure 1). The three high-yield strains adopted in this study are excellent and practical strains, which are widely used in breeding [19,20,21], and the average CSW is about three times of the selected low-yield strains (Table 1). We investigated the traits of whole cocoon weight, CSW, and pupa weight of 12 silkworm strains, including sequenced strains of males and females (Table 1). Significant differences in cocoon traits were observed among strains. CSW was about four-fold higher in the high-yield strains than in the low-yield strains. In addition, the high-yield strains had two- to three-fold higher cocoon shell rates than the low-yield strains. Silk glands showed different development rates among strains and developmental stages. The developmental rate of the silk glands of strain 19-200 peaked on the day before mounting (Figure 2). We collected samples at this time point to ensure that the majority of genes associated with silk traits were upregulated. Furthermore, different silkworm silk glands have different lengths of development. The accurate selection of the dissection time-point is thus critical to ensure that the silk glands of each strain are at the same stage of development. Therefore, to ensure accuracy of the sequencing data, the fifth instar (5.5 days) of strain 19-200 was used as the standard, and its length was then used in calculating the dissection time-point (Table 2) of each strain.

### 2.2. Overview of Transcriptome Sequencing Data

For each strain, we randomly selected three larvae, from which the entire silk gland was isolated and RNA was extracted. RNA sequencing was performed using the Illumina HiSeq^TM^ 4000 system [22]. After filtering the data for adaptor sequences, unknown sequences (N), and low-quality reads, 45.9 gigabases (Gb), 46.8 Gb, and 45.1 Gb, 46.5 Gb, 44.7G, and 46.9G of clean reads were obtained for strains, 10-710, 19-200, 19-460, 872B, Xia Fang, and Qiu Bai, respectively. We compared the clean reads with the silkworm reference genome, and the percentage of total reads in the six transcriptome databases ranged from 52.1% to 72.3% (Table 3). The average number of clean reads was about 46 million. Among all strains, 29.6%–40.84% of the clean reads were aligned to about 9,000 genes, of which strain 872B had the highest number of genes, in which 9,397 reads could be aligned to the silkworm reference genome (Table S1). The number of new transcripts for each strain ranged from 842 to 981 (Table S2).

### 2.3. Differential Expressed Gene Function Annotation and Functional Analysis

**Figure 3:**
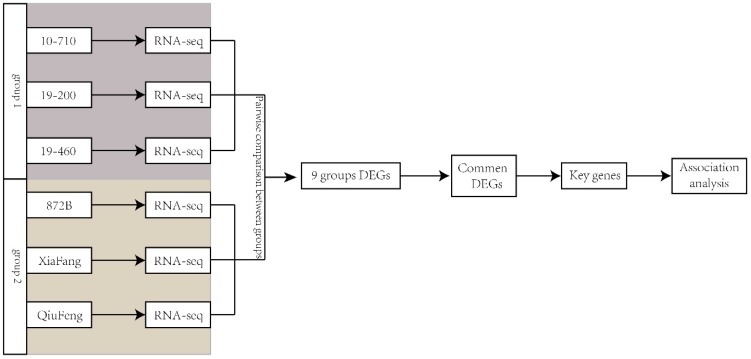
Experimental comparison strategy design. By comparing the three high-yield strains with the three low-yield strains, nine groups of DEGs were obtained. Differentially expressed in more than four of the nine groups of DEGs were defined as common DEGs. The key genes were obtained by screening the common DEGs through quantitative detection of each strain in silk glands. Finally, the association analysis with the cocoon shell weight (CSW) was conducted in key genes.

The expression of all genes was estimated using the RPKM (reads per kb per million reads) method [23]. The following comparison strategy for screening DEGs was adopted in this study (Figure 3). By comparing the three high-yield strains with the three low-yield strains, nine groups of DEGs were obtained. The edgeR function in Bioconductor was employed for differential expression analysis [24]. All the DEGs are presented in Table 4. The number of DEGs in each group varied from 8 to 261 (Table S3). The gene sequences were aligned to the NR (Non-Redundant Protein Sequence Database) library using BLAST to extract DEG annotation information. The topGO function in the software, Bioconductor, was employed for DEG enrichment analysis [25].

For functional annotation of the DEGs, GO enrichment analysis was performed. GO terms with a corrected *p* < 0.05 were considered significantly enriched with DEGs. GO functional enrichment analysis of DEGs of the six strains revealed significant enrichment in the categories of biological process and molecular function. Further assessment revealed that the annotation information of nine DEGs were similar. In the functional category of biological process, significant DEG enrichment between the high- and low-yield strains was observed for the subcategories of cellular process, metabolic process, and single-organism process. For the functional category of cellular components, DEG enrichment was also detected in the subcategories of extracellular and extracellular regions. In terms of molecular function, DEG enrichment was observed in the subcategories of binding and catalytic activity (Figure S1). BLAST analysis of the DEGs to the KEGG database was performed to obtain annotation information relating to pathways [26]. Based on the resulting list of DEGs, a hypergeometric test was used to perform pathway enrichment analysis of DEGs, which showed that the endoplasmic reticulum pathway in protein processing of metabolic pathways was enriched with DEGs between the high-and low-yield silkworms (Figure S2).

### 2.4. Quantitative Verification of DEGs and Multistrain Association Analysis

**Figure 4:**
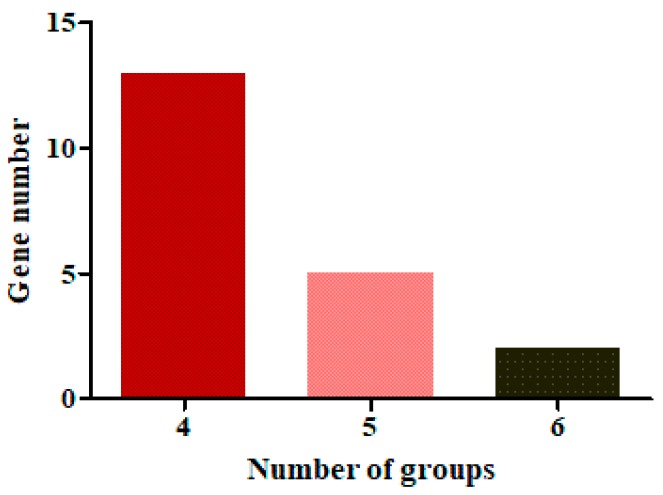
The statistics of common DEGs among groups. The abscissa indicates the number of groups with the same DEGs. The ordinate shows the number of common DEGs in the various numbers of groups.

Figure 4 shows the DEGs identified from the comparison of multiple groups. Twenty genes that were differentially expressed in more than four of the nine groups of DEGs were defined as common DEGs (Table 4), which included nine genes that were upregulated in low-yield strains, and 11 genes that were upregulated in high-yield strains. Five of the 20 common DEGs were detected in at least five groups of BLAST data.

**Figure 5:**
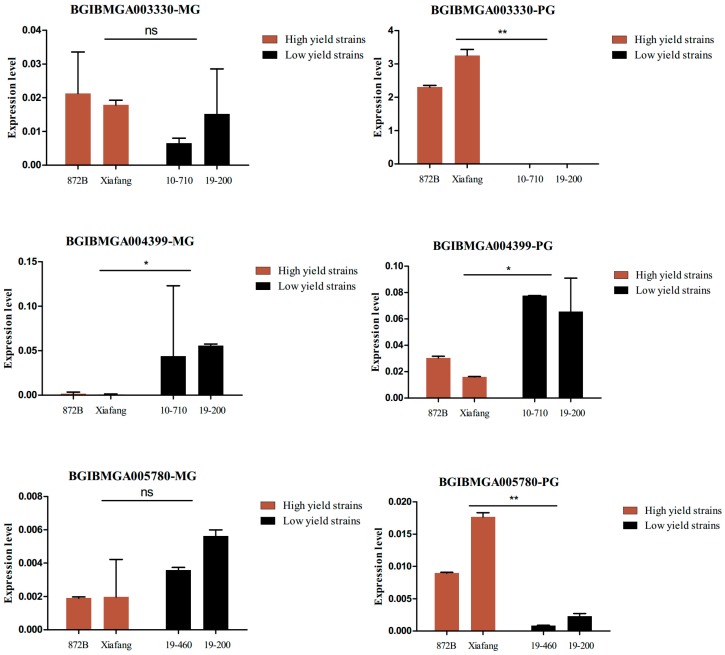

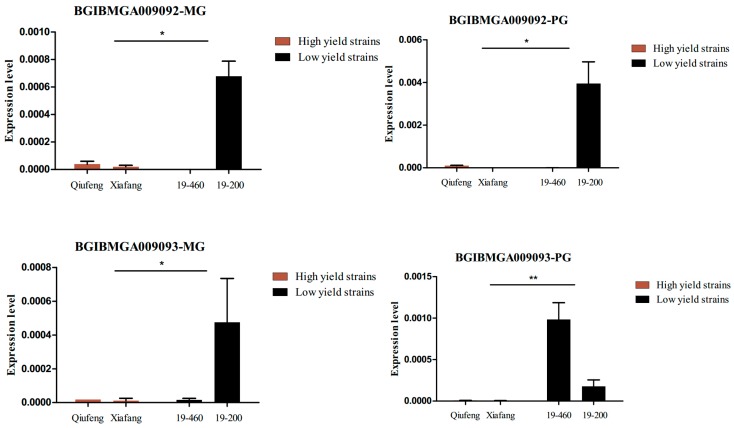
Quantitative verification of common DEGs. MG: Middle silk gland, PG: Posterior silk gland. ns: Not significant; *: *p* ≤ 0.05; **: *p* ≤ 0.01.

**Figure 6:**
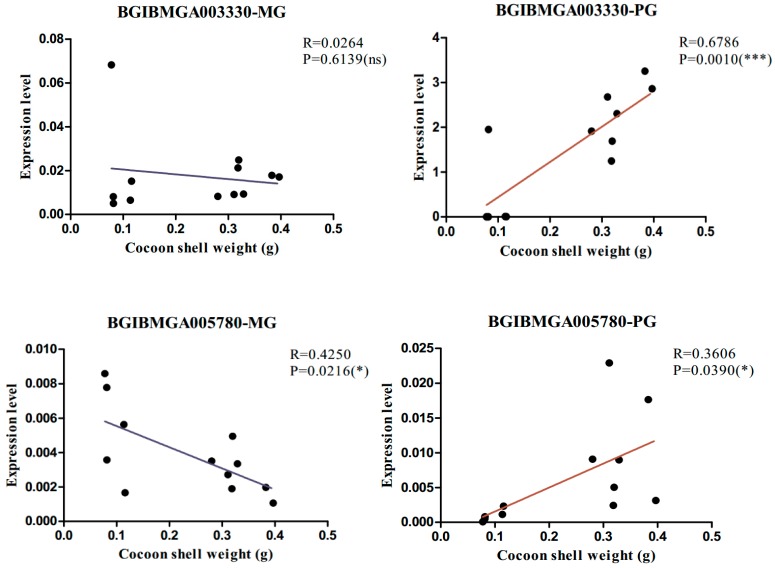
Association analysis of partial gene expression levels with cocoon shell weight (CSW). In the figure, only the association analysis diagram of the associated significant gene is listed, ns: Not significant; *: *p* ≤ 0.05; ***: *p* ≤ 0.001.

We validated five significant common DEGs in the MGs and PGs of the strains that were used for sequencing by qPCR (Figure 5), and the results coincided with the RNA-seq data. *BGIBMGA004399*, *BGIBMGA009092*, and *BGIBMGA009093* genes in the MGs, and *BGIBMGA005780* and *BGIBMGA003330* genes in the PGs were differentially expressed between the high- and low-yield strains. We further analyzed the association between the five significant common DEGs and silk yield traits in the 12 strains with different silk yields by qPCR (Figure S3 and Figure 6), which indicated that the expression of BGIBMGA003330 and BGIBMGA005780 in the silk gland is closely related to the silk fibroin generated (Figure 6).

### 2.5. PCA of Wild Silkworm, Local Strains, and Practical Strains

**Figure 7:**
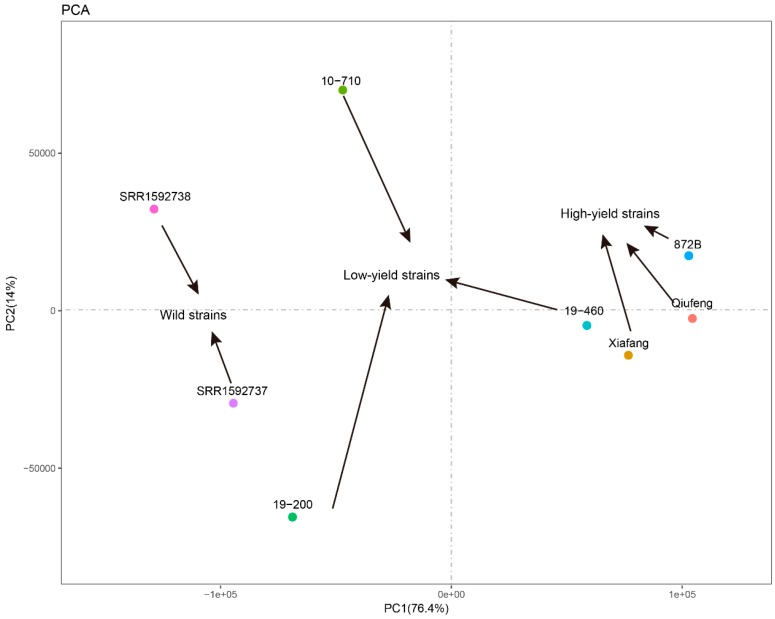
Principal component analysis (PCA). The numbers 1, 2, and 6 represent high-yield strains; 3, 4, and 5 indicate low-yield strains; and w1 and w2 are wild silkworms. The wild silkworm transcriptome data, w1 and w2, were derived from a comparative analysis of the silk gland transcriptomes between the domestic and wild silkworms [Fang et al. BMC Genomics (2015)].

Cocoon quality traits undergo two stages of selection, namely, the domestication process from wild silkworm strains to local strains, and the breeding process from local species to practical species. PCA was employed to detect differences in gene expression patterns among various strains by analyzing similarities in major strain components. Thus, differences in selection for silkworm cocoon quality traits in these two processes can be inferred. In this study, the data of three local strains and three practical strains were combined with two wild silkworm data from other studies [16] to conduct PCA. The results showed that the main components of the two wild silkworm strains and the three local strains were distributed, whereas those of the practical strains were clustered together (Figure 7). Compared to the domestication process, modern breeding imparts stronger selection pressure on genes that are related to silk yield, so that silkworms of different high-yield strains exhibit similar expression patterns. The common genes that are highly expressed in the three high-yield silkworm strains are likely to be important genes that control silk yield. Based on these results, we identified genes that are highly expressed in all three high-yield strains, followed by functional annotation (Table 4). Most of these genes are ribosomal proteins and structural proteins, which presumably play an important role in the biosynthesis of silk protein.

## 3. Discussion

In this study, multiple silkworm strains with different cocoon colors were used as research materials. By screening the common DEGs, the background interference caused by the particularity of a single strain, such as the genes controlling cocoon color, could be better avoided, making the results more accurate and reliable. RNA-seq analysis of various silkworm strains identified up to 261 DEGs in each pair of samples, and 22 common DEGs were screened. These common DEGs mostly function in energy metabolism and protein synthesis. The results suggest that the energy metabolic rate is related to silk gland development. In addition, protein synthesis affects silk protein synthesis efficiency, thus affecting silkworms’ silk production.

Common DEGs were identified by association analysis, and gene BGIBMGA003330 and BGIBMGA005780 were significantly associated with CSW. From the results of quantitative verification, we can see that the expression levels of genes BGIBMGA003330 and BGIBMGA005780 presented significant differences between the high-yield and low-yield strains in the PGs. The PGs are used to produce silk fibroin. Silk sericin, wrapped in the outer layer of silk fibroin to protect and bind it, is water-soluble and soluble in water, acid, and alkali solutions. However, silk fibroin only swells in water, and is insoluble in water. In the process of production and utilization, silk fibroin is separated by sericin melting during the process of filature, which is the main material for silk products. It can be seen that the increase of silk fibroin production is the core of the increase of silk yield. We speculate that BGIBMGA003330 and BGIBMGA005780 may be related to silk fibroin synthesis. Genes BGIBMGA003330 and BGIBMGA005780 encode uridine nucleosidase and small heat shock protein (sHSP), respectively. Uridine nucleosidase plays a fundamental role in the interconversion of pyrimidine nucleotides and in the reutilization of pyrimidine nucleosides. Uridine nucleosidase is used to decompose uridine into uracil and ribose [27], then uracil is incorporated into the mononucleotide, UMP (Uridine monophosphate) [28]. UMP is the composition of nucleic acid involved in the basic life activities of organisms, such as heredity, development, and growth [29]. Ribose, of fundamental importance in the pyrimidine nucleotides’ biosynthesis from exogenously supplied or endogenously formed bases and nucleosides, is involved in the biosynthesis of storage compounds and the synthesis of structural building compounds [30]. The role of this gene in development and protein synthesis may be related to the development of silk glands or silk protein synthesis, thereby affecting the silk yield.

Small heat shock protein is a molecular chaperone with strong anti-aggregation properties. Previous studies have shown that sHSP enhances heat tolerance [31], protects cells from stress [32], prevents protein misfolding [33], restores the natural concept of unfolded or partially folded peptides [34], alters mitochondrial metabolism [35], controls mitochondria protein quality to extend the lifespan [36], influences the rate of embryonic development, and prevents spontaneous diapause [37]. sHSP are not only expressed independently in the heat shock response, but also play an important role in some animal development processes, which may be related to silk gland development, such as regulating cell movement [38]; regulating intermediate filament assembly [39]; inhibiting apoptotic signaling [40]; binding to certain kinases to activate and protect them from heat inactivation [41]; and allowing cancerous cells to escape the immunosurveillance mediated by death ligands [42]. There is no evidence related to silk gland development in the current report, but in our study, it was found that this gene is significantly associated with the silkworm CSW. Moreover, there is also evidence that sHSP maintains protein structure and enhance the growth of tumors in vivo [42]. So, it is also possible that these may also have other functions that are related silk gland development that have yet to be discovered.

Besides, the common DEGs, BGIBMGA004399 and BGIBMGA008165, encode a 30K protein that is involved in embryonic development [43], energy storage [44], and the immune response [45] in the silkworm, plays an important role in energy metabolism, and contributes to inhibiting apoptosis [46]. We postulate that these genes affect the rate of silk protein synthesis by regulating energy metabolism, thereby affecting silk yield traits.

In this study, PCA results attracted our attention. PCA indicated that low-yield strains significantly vary in terms of gene expression patterns, whereas high-yield strains have highly similar expression patterns (Figure 6). Previous studies have shown that the linkage groups that control the QTLs for different parents overlap [47,48,49,50], suggesting that unlike the long domestication process, modern genetic breeding has a strong selective effect on silk yield traits. The rapid accumulation of superior genes for cocoon traits or the generation of new genes that increase yield can lead to higher breeding values in the short term. Genes that are highly expressed in the three high-yield strains are likely to be important genes that control silk yield. We have identified that genes that are highly expressed in the three high-yield strains are relatively expressed at low levels in the three low-yield strains, and were functionally annotated. Most of these genes are ribosomal proteins and structural proteins, which may play an important role in the biosynthesis of silk protein.

In summary, we have described an approach to circumvent the effect of background differences using multi-strand RNA-seq alignments to more accurately screen for genes related to silk yield. The differentially expressed genes identified in this study may serve as the object of the functional study, and it is necessary to deeply study its function and the mechanism of affecting silk yield.

## 4. Materials and Methods

### 4.1. Silkworm Breeding and Sample Preparation

The selected materials were six silkworm strains for RNA-seq, including three high-yield strains (Qiufeng, Xiafang, and 872B), and three low-yield strains (10-710, 19-200, and 19-460), and 12 strains (Table 1) with different cocoon traits and silk yields for correction analysis. The silkworms were obtained from the silkworm resource bank of Southwest University, Chongqing, China. The larvae were fed fresh mulberry leaves and maintained at conditions of sTable 14 h light and 10 h dark photoperiod at 25 ± 1 °C and 75% ± 3% RH. Intact silk glands were dissected and frozen immediately in liquid nitrogen and then stored at −80 °C for sequencing. In this study, three samples were prepared as biological replicates.

The silkworm cocoons of different strains were collected for phenotypic assessment, and these silkworms were reared at the same time and in the same conditions. The cocoons of the low-yield silkworms (19-460, 10-710, and 19-200) were much smaller than the high-yield strains (872B, Qiufeng, and Xiafang). The statistical results of CSW show significant differences between the low- and high-yield strains.

### 4.2. RNA Library Construction and Sequencing

Total RNA was extracted using TRIzol reagent according to the manufacturer’s recommendations (Invitrogen, Burlington, ON, Canada), and DNA was digested with DNase I. Eukaryotic mRNAs are enriched with oligo(dT) magnetic beads. Using a thermomixer, the mRNAs were sheared into short fragments under the appropriate temperature, and a strand of cDNAs were synthesized using the interrupted mRNAs as template. Then, a two-stranded synthesis reaction system was used to synthesize the two-stranded cDNA, and kits were used for purification and recovery, cohesive end-repair, adding the base “A” to the 3′ end of the cDNAs, and attaching the adapter, followed by fragment size selection and PCR amplification. The constructed library was qualified with the Agilent 2100 Bioanalyzer and the ABI StepOne Plus Real-Time PCR system and then sequenced using the Illumina HiSeq^TM^ 4000. The data from the Illumina HiSeq^TM^ 4000 sequence were designated as raw reads or raw data [22], which were then subjected to quality control (QC) to determine that the sequencing data is suitable for subsequent analysis. After QC, the raw reads were filtered to obtain clean reads, and a SOAP aligner/SOAP2 [51] was used to compare the clean reads to the reference sequence. A BLAST search was then performed to determine the distribution and coverage of reads on the reference sequence to assess whether the comparison results passed the second QC of alignment, thereby ensuring that subsequent analysis is based on high-quality clean reads.

Reference sequence and gene model annotations were downloaded from the Silkworm Genome Database (SilkDB; http://www.silkdb.org/silkdb/) [52]. We compared the clean data of each sample with the reference gene set of the silkworm using the short reads software, SOAPaligner/SOAP2 [51], and compared the clean data of each sample with the reference genome using TopHat (http://ccb.jhu.edu/software/tophat/index.shtml) [53]. The results were then used in the analyses for alternative splicing and prediction of new transcripts.

### 4.3. Quantitative and Differential Analysis of Transcripts

We used the reads per kilobase per million reads (RPKM) [23] method to estimate gene expression levels. The differential expression of two samples was screened using the edgeR [24] function in Bioconductor. The edgeR function assumes that the count of sequencing reads is a negative binomial distribution for each gene. Hypothesis testing was performed based on this theoretical distribution. The differentially expressed gene screening conditions were set to FDR ≤ 0.05 |log_2_ratio| ≥ 1 [54]. Genes with similar expression patterns usually have similar functions. To analyze gene expression patterns, we used the R software and used the Euclidean distance as the distance calculation formula to perform hierarchical clustering of DEGs and experimental conditions.

### 4.4. Differential Gene Function Annotation and Functional Analysis

We obtained the reference sequence for all silkworm genes from the NCBI database and used BLASTX for protein annotation. Then, we employed the BLAST software to align the gene sequences to the NR library and extracted the GO annotation information for all genes from the Gene Ontology database (http:/www.geneontology.org/). Based on the list of DEGs, we performed GO enrichment analysis of DEGs using the topGO function in the software, Bioconductor [25]. The KEGG analysis [26] involves a BLAST search of genes in the KEGG database to annotate pathway information on the DEGs. Then, based on the list of DEGs obtained, the pathway enrichment analysis of DEGs was performed using hypergeometric testing to find the pathway that was significantly enriched with DEGs compared to the entire genomic background.

### 4.5. Expression Quantity Identification of DEGs

To verify RNA-seq data, we used real-time quantitative PCR (qPCR) to identify DEGs for expression quantity identification. Intact silk glands were dissected according to the timetable for sample preparation. In addition, other tissues of the fifth instar 3-day larvae of 19-200 and 872B, including the head, epidermis, midgut, Malpighian tubule, fat body, hemolymph, middle silk gland, posterior silk gland, trachea plexus, testis, nerves, and ovaries, were dissected for tissue expression identification of DEGs. Total RNA was extracted using the RNApure ultrapure total RNA Rapid Extraction Kit (BioTek) strictly following the manufacturer’s instructions. Ultraviolet spectrophotometry was used to determine RNA integrity and purity. The cDNA was extracted using the PrimeScript RT reagent kit with a gDNA Eraser kit, and cDNA purity was assessed by PCR using primers specific to Actin3 (A3), a silkworm housekeeping gene. The CDS sequences of the confirmed genes were extracted from the Silkworm Genome Database (http://www.silkdb.org/silkdb/) [42] and used in designing primers to span at least one intron. qPCR was performed using the CFX96TM Real-Time PCR Detection System (Bio-Rad, Hercules, CA, USA) and SYBR Green qPCR Mix (Bio-Rad) reagents. qPCR was conducted in a reaction volume of 10 μL containing 1 μL of the template, 5 μL of 2 × SYBR Green II, 5 μL of ddH_2_O, and 0.3 μL of the specific primers. The PCR conditions were as follows: Pre-denaturation at 95 °C for 30 s; followed by 40 cycles of denaturation at 95 °C for 5 s and annealing at 60 °C for 30 s; and a melting curve at 65 °C for 5 s, with a stepwise temperature increase of 0.5 °C until 95 °C. The volume of the reaction system was 10 μL, and three technical replicates were used per sample. The expression levels of each gene of the two strains were compared based on a *t*-test. Differences in gene expression between high- and low-yield strains were considered significantly different at *p* < 0.05.

## Figures and Tables

**Table 1:** Statistical analysis of yield-related traits in various silkworm strains.

Strain	Whole Cocoon Weight (g)		Cocoon Shell Weight (g)		Cocoon Shell Rate		Pupa Weight (g)	
	Males	Females	Males	Females	Males	Females	Males	Females
19-460	0.579 ± 0.016	0.772 ± 0.032	0.074 ± 0.003	0.082 ± 0.005	0.127 ± 0.004	0.106 ± 0.004	0.505 ± 0.014	0.691 ± 0.029
19-450	0.611 ± 0.03	0.75 ± 0.057	0.078 ± 0.005	0.082 ± 0.007	0.128 ± 0.006	0.109 ± 0.005	0.532 ± 0.027	0.668 ± 0.051
10-710	0.656 ± 0.029	0.848 ± 0.035	0.08 ± 0.005	0.084 ± 0.004	0.123 ± 0.004	0.099 ± 0.005	0.575 ± 0.025	0.763 ± 0.034
05-036	0.626 ± 0.024	0.799 ± 0.051	0.112 ± 0.006	0.122 ± 0.011	0.179 ± 0.005	0.152 ± 0.006	0.514 ± 0.018	0.677 ± 0.041
19-200	0.714 ± 0.038	0.928 ± 0.07	0.103 ± 0.009	0.118 ± 0.016	0.143 ± 0.008	0.126 ± 0.009	0.612 ± 0.031	0.81 ± 0.055
HB	1.115 ± 0.038	1.338 ± 0.083	0.276 ± 0.012	0.276 ± 0.022	0.248 ± 0.006	0.206 ± 0.007	0.839 ± 0.03	1.062 ± 0.064
Qiubai	1.296 ± 0.038	1.565 ± 0.05	0.304 ± 0.013	0.315 ± 0.012	0.235 ± 0.009	0.201 ± 0.006	0.992 ± 0.034	1.25 ± 0.043
Qiufeng	1.233 ± 0.05	1.482 ± 0.065	0.308 ± 0.013	0.326 ± 0.013	0.25 ± 0.008	0.22 ± 0.009	0.925 ± 0.042	1.157 ± 0.059
7532	1.349 ± 0.111	1.643 ± 0.036	0.312 ± 0.011	0.329 ± 0.012	0.232 ± 0.015	0.2 ± 0.004	1.037 ± 0.106	1.315 ± 0.026
872B	1.307 ± 0.034	1.558 ± 0.045	0.329 ± 0.009	0.331 ± 0.016	0.252 ± 0.009	0.213 ± 0.006	0.978 ± 0.036	1.227 ± 0.033
871B	1.379 ± 0.038	1.806 ± 0.066	0.364 ± 0.007	0.428 ± 0.023	0.264 ± 0.006	0.237 ± 0.006	1.016 ± 0.034	1.377 ± 0.045
Xiafang	1.488 ± 0.066	1.796 ± 0.056	0.375 ± 0.014	0.391 ± 0.01	0.252 ± 0.008	0.218 ± 0.004	1.113 ± 0.057	1.404 ± 0.048

**Table 2:** Dissection time points of different strains.

Strain	Time of the Fifth Instar (days)	The Time Point of Silk Gland Dissected (day)
10-710	7.5	6.5
19-200	6.5	5.5
19-460	5.5	4.5
872B	8.5	7.5
Xiafang	7.5	6.5
Qiufeng	8.0	7.0

**Table 3:** Summary of the sequence assembly after lllumina sequencing.

Strain	Number of Raw Reads	Number of Clean Reads	Number of Clean Bases	Q20 (%)	GC Content (%)
10-710	54,026,974	45,861,944	6,879,291,600	96.14	50.69
19-200	54,023,574	46,787,486	7,018,122,900	97.07	49.31
19-460	54,024,582	45,123,438	6,768,515,700	96.51	51.19
872B	55,663,714	46,508,788	6,976,318,200	96.19	51.96
Xiafang	56,894,572	44,654,180	6,698,127,000	95.78	52.37
Qiufeng	57,300,696	46,940,002	7,041,000,300	95.92	53.00

**Table 4:** Functional annotation of the common DEGs in high-yield vs. low-yield silkworms.

Gene ID	Chromosome	Regulation (High-/Low-Yield Strains)	Gene Annotation
*BGIBMGA001700*	11	Down	Ubiquitin-related modifier 1 homolog [*Bombyx mori*]
*BGIBMGA001816*	11	Up	Uncharacterized protein LOC101745939 [*Bombyx mori*]
*BGIBMGA002441*	9	Up	gi|512926587|ref|XP_004931123.1|/2.3936e-58/PREDICTED: uncharacterized protein LOC101741881 [*Bombyx mori*]
*BGIBMGA002629*	5	Down	gi|237648976|ref|NP_001153665.1|/4.06608e-85/odorant binding protein LOC100301497 precursor [*Bombyx mori*]
*BGIBMGA005780*	5	Up	gi|512896232|ref|XP_004923862.1|/2.0943e-140/PREDICTED: protein lethal (2) essential for life-like [*Bombyx mori*]
*BGIBMGA014291*	5	Down	gi|827563795|ref|XP_004933964.2|/6.52866e-100/PREDICTED: alcohol dehydrogenase-related 31 kDa protein-like, partial [*Bombyx mori*]
*BGIBMGA003230*	2	Up	gi|930671460|gb|KPJ12187.1|/0/RNA-directed RNA polymerase L [*Papilio machaon*]
*BGIBMGA003330*	15	Up	gi|512892835|ref|XP_004923030.1|/0/PREDICTED: probable uridine nucleosidase 2 [*Bombyx mori*]
*BGIBMGA004397*	20	Down	gi|525342977|ref|NP_001266309.1|/0/low molecular mass 30 kDa lipoprotein 19G1-like precursor [*Bombyx mori*]
*BGIBMGA004399*	20	Down	gi|379046488|gb|AFC87805.1|/0/30K protein 7 [*Bombyx mori*]
*BGIBMGA004614*	27	Up	gi|827548595|ref|XP_012546487.1|/0/PREDICTED: heat shock protein 70 A2-like [*Bombyx mori*]
*BGIBMGA007353*	3	Up	gi|827541356|ref|XP_012543946.1|/0/PREDICTED: uncharacterized protein LOC105841300 [*Bombyx mori*]
*BGIBMGA008677*	7	Up	gi|913296126|ref|XP_013183364.1|/9.54277e-117/PREDICTED: uncharacterized protein LOC106129371 [*Amyelois transitella*]
*BGIBMGA008712*	7	Up	gi|827546128|ref|XP_012545526.1|/0/PREDICTED: Bardet-Biedl syndrome 5 protein homolog [*Bombyx mori*]
*BGIBMGA010133*	7	Down	gi|512927483|ref|XP_004931346.1|/0/PREDICTED: regucalcin-like [*Bombyx mori*]
*BGIBMGA009092*	3	Down	gi|827031951|gb|AKJ54535.1|/4.53413e-66/fungal protease inhibitor [*Bombyx mori*]
*BGIBMGA009093*	3	Down	gi|512898429|ref|XP_004924398.1|/1.63675e-30/PREDICTED: zonadhesin-like isoform X1 [*Bombyx mori*]
*BGIBMGA010892*	22	Down	gi|827542143|ref|XP_012544229.1|/4.51314e-92/PREDICTED: zonadhesin-like isoform X1 [*Bombyx mori*]
*BGIBMGA013130*	16	Up	gi|512909270|ref|XP_004926880.1|/0/PREDICTED: glucosylceramidase-like [*Bombyx mori*]
*BGIBMGA013477*	6	Up	gi|512908146|ref|XP_004926609.1|/0/PREDICTED: synaptic vesicle glycoprotein 2C-like [*Bombyx mori*]

## Supplementary Materials

Supplementary materials can be found at http://www.mdpi.com/1422-0067/19/12/3718/s1.

## Abbreviations

DEGs	Differentially expressed genes
PCA	Principal component analysis
QTL	Quantitative trait loci
CSW	Cocoon shell weight
