# Supplementary figures and images for: Identification of Genes that Control Silk Yield by RNA Sequencing Analysis of Silkworm (Bombyx mori) Strains of Variable Silk Yield
Source: Int J Mol Sci. 2018 Nov 22;19(12):3718. doi: 10.3390/ijms19123718 (PMC6321331; doi:10.3390/ijms19123718)

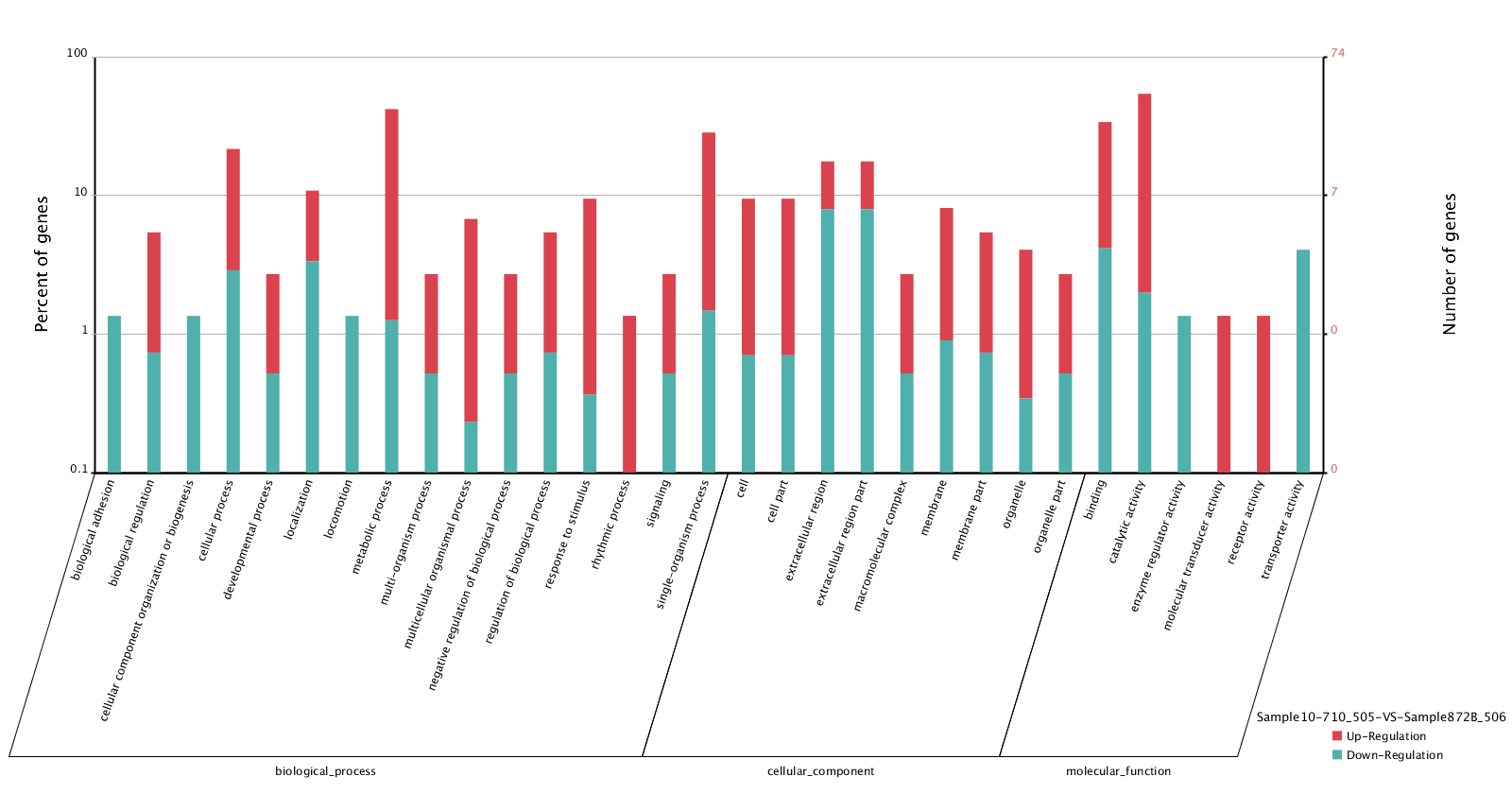

Supplement: Supplementary file 1 [file ijms-19-03718-s001.zip › Figure and Table/Figure S1/Sample10-710_505-VS-Sample872B_506.goclass.png]

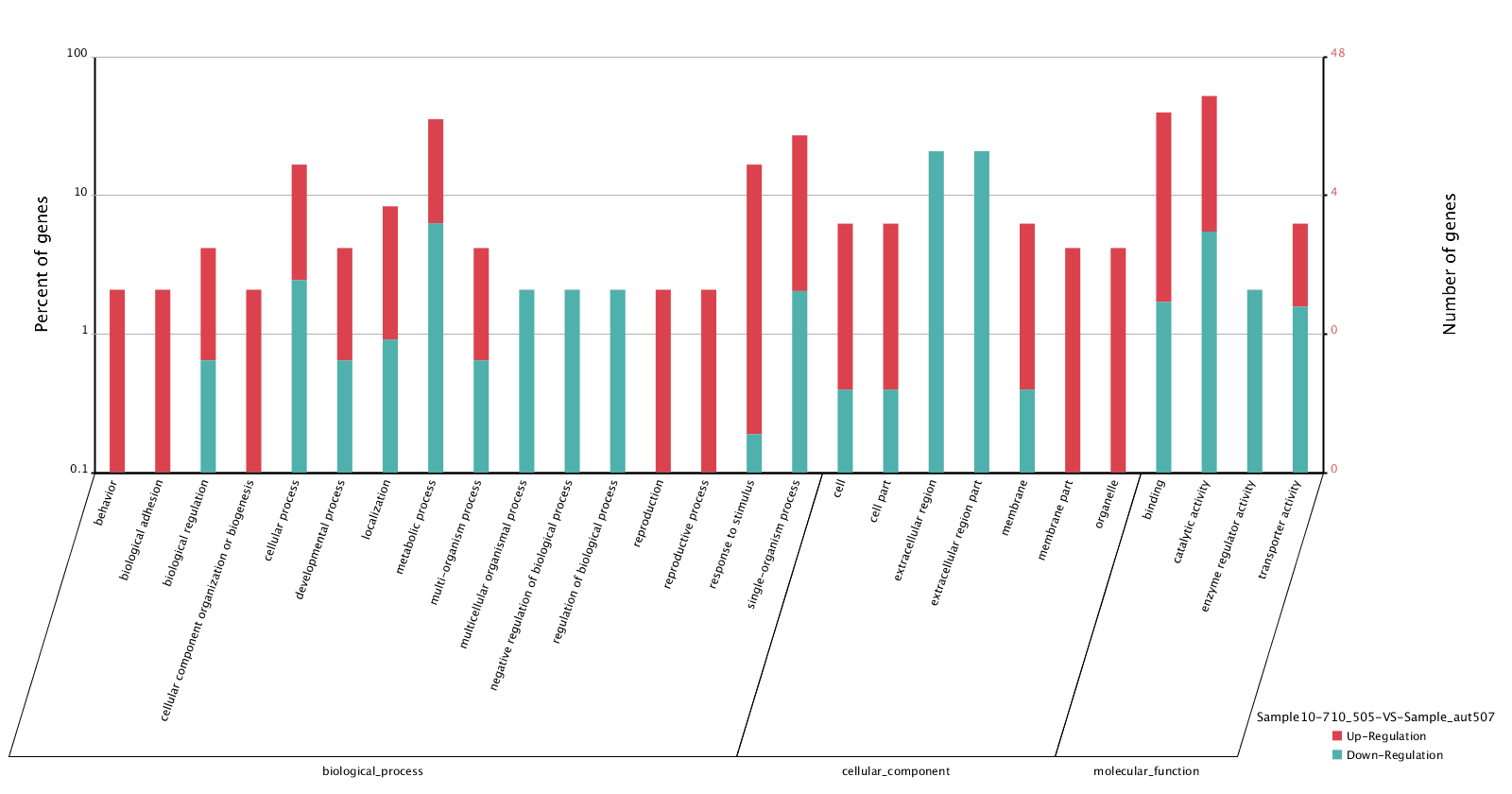

Supplement: Supplementary file 1 [file ijms-19-03718-s001.zip › Figure and Table/Figure S1/Sample10-710_505-VS-Sample_aut507.goclass.png]

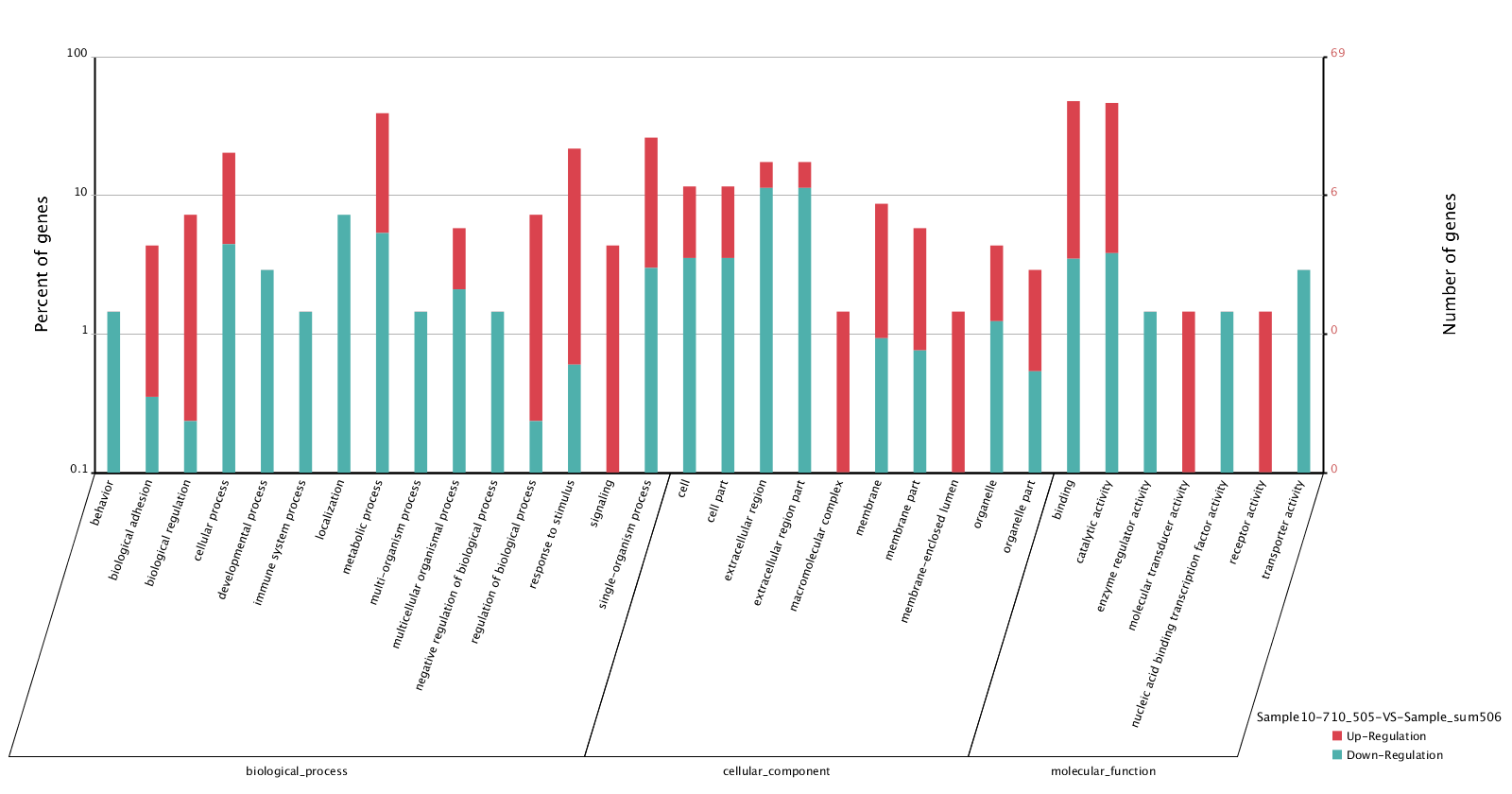

Supplement: Supplementary file 1 [file ijms-19-03718-s001.zip › Figure and Table/Figure S1/Sample10-710_505-VS-Sample_sum506.goclass.png]

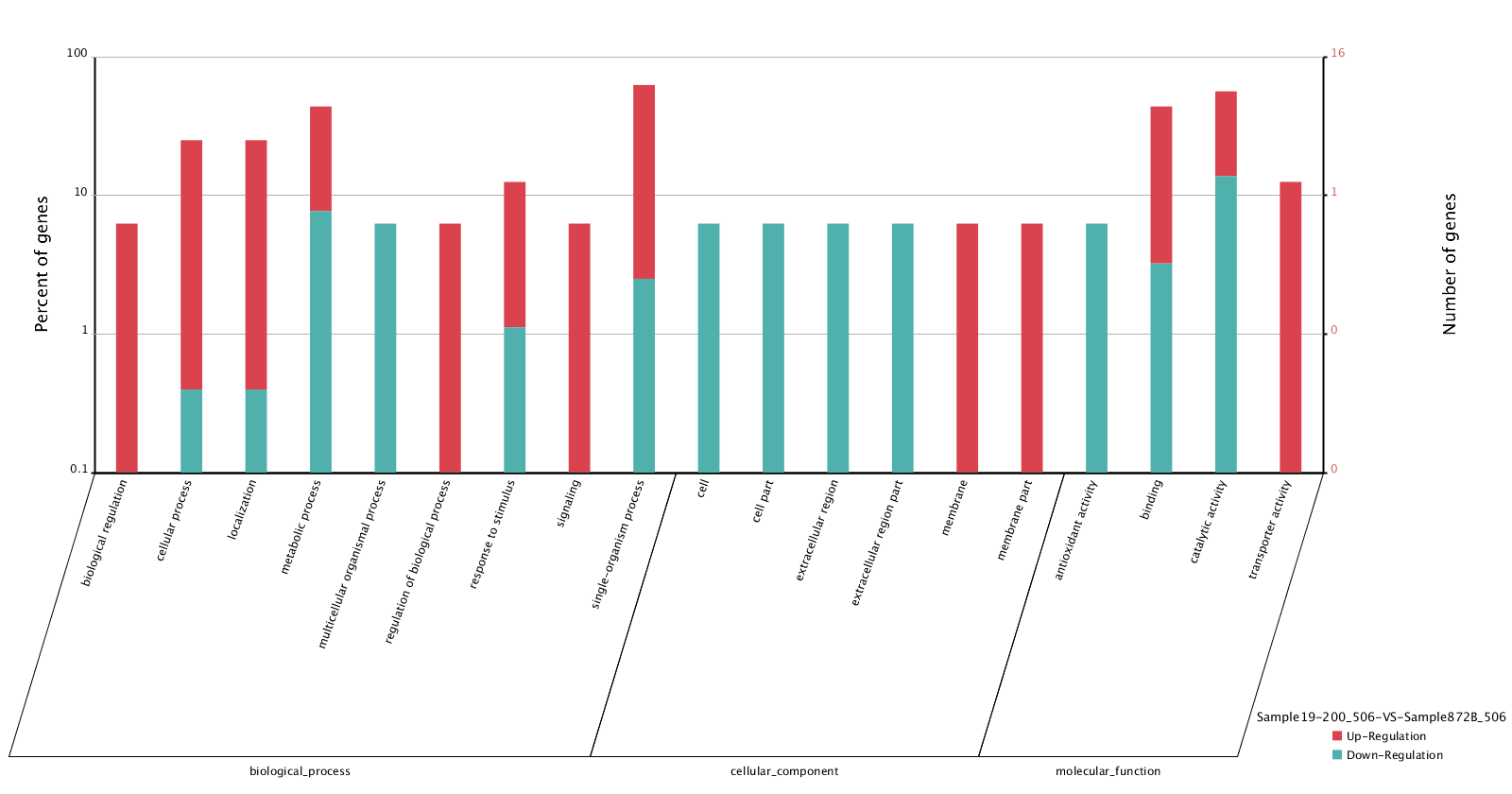

Supplement: Supplementary file 1 [file ijms-19-03718-s001.zip › Figure and Table/Figure S1/Sample19-200_506-VS-Sample872B_506.goclass.png]

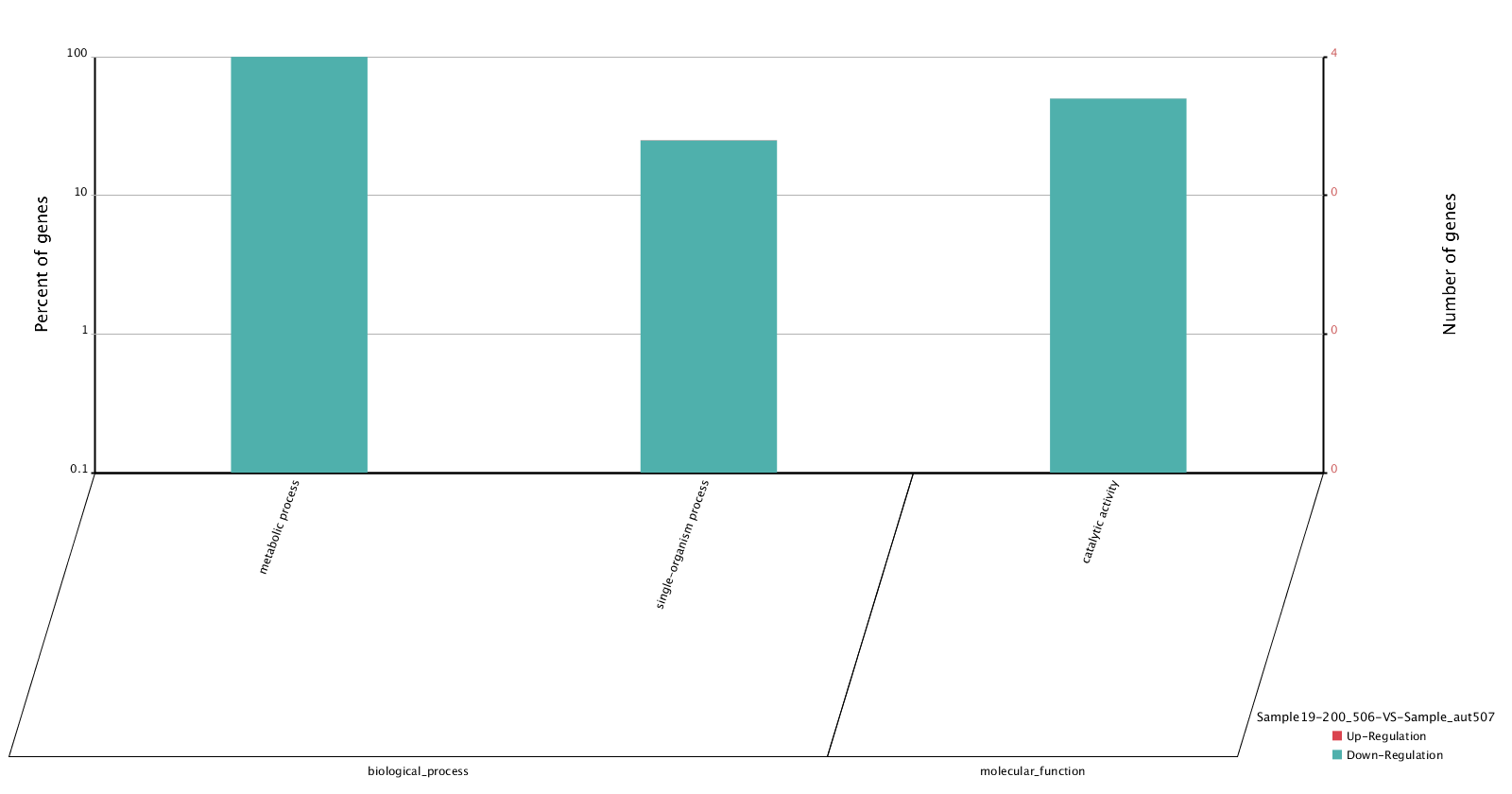

Supplement: Supplementary file 1 [file ijms-19-03718-s001.zip › Figure and Table/Figure S1/Sample19-200_506-VS-Sample_aut507.goclass.png]

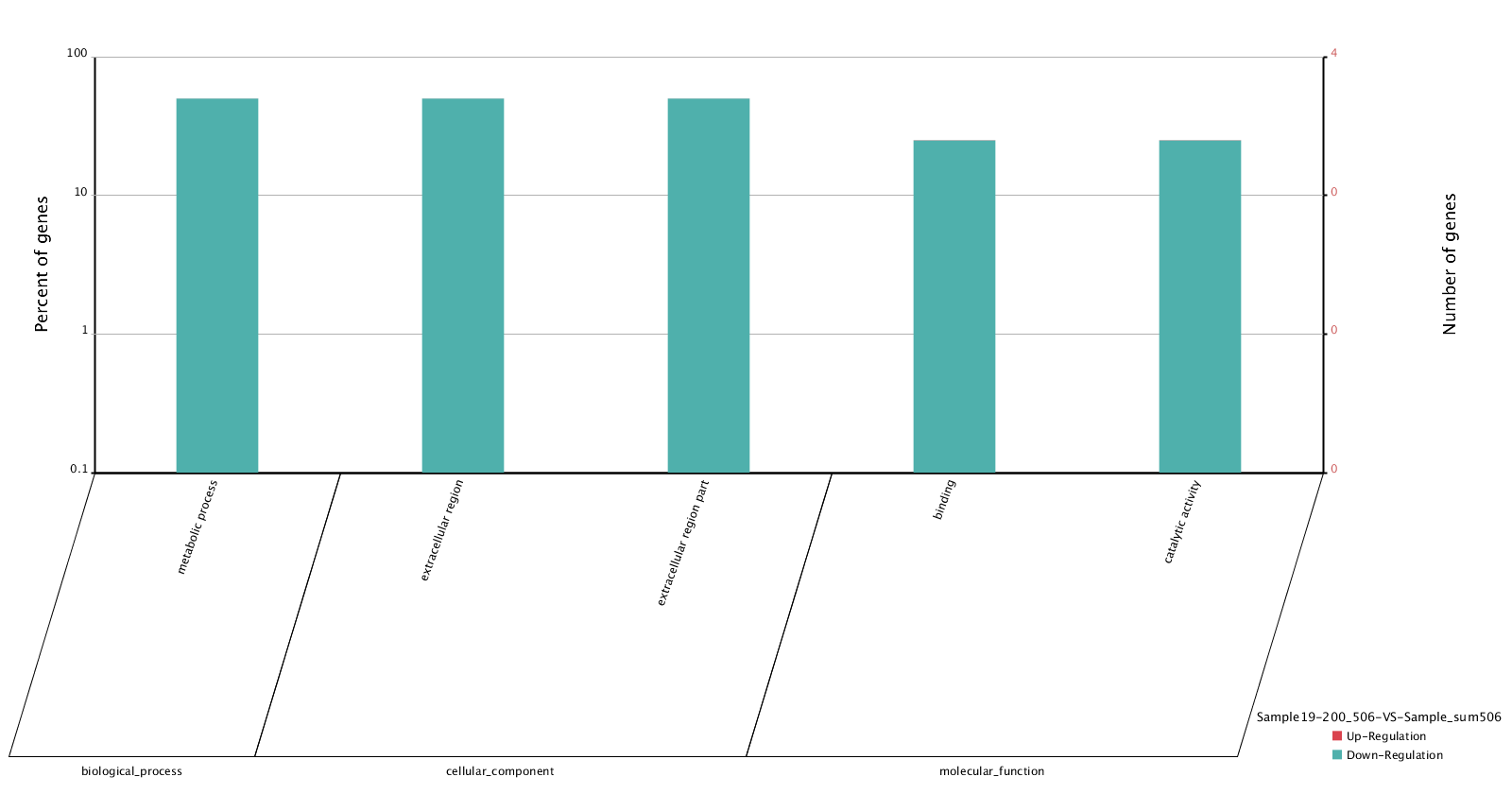

Supplement: Supplementary file 1 [file ijms-19-03718-s001.zip › Figure and Table/Figure S1/Sample19-200_506-VS-Sample_sum506.goclass.png]

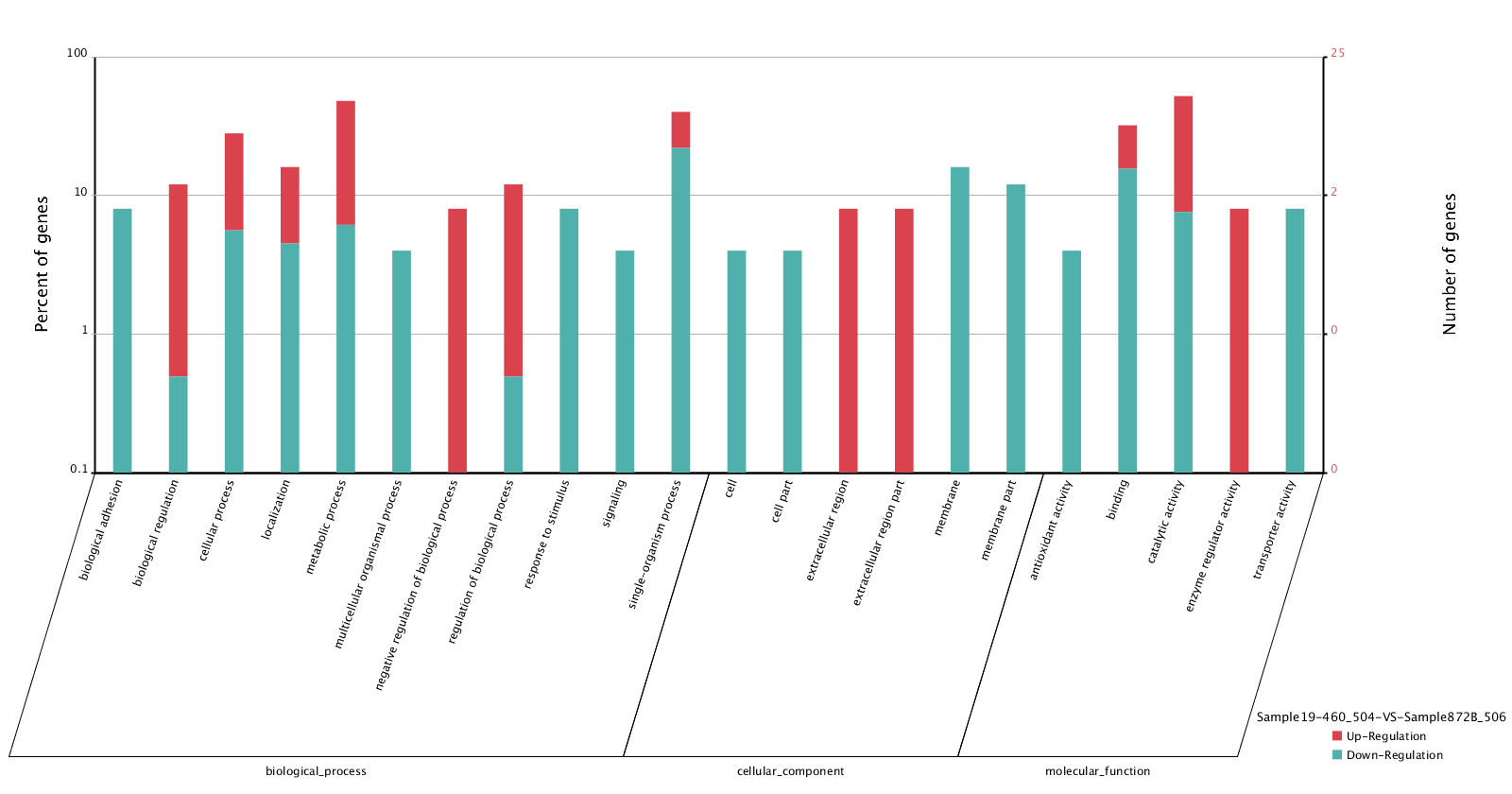

Supplement: Supplementary file 1 [file ijms-19-03718-s001.zip › Figure and Table/Figure S1/Sample19-460_504-VS-Sample872B_506.goclass.png]

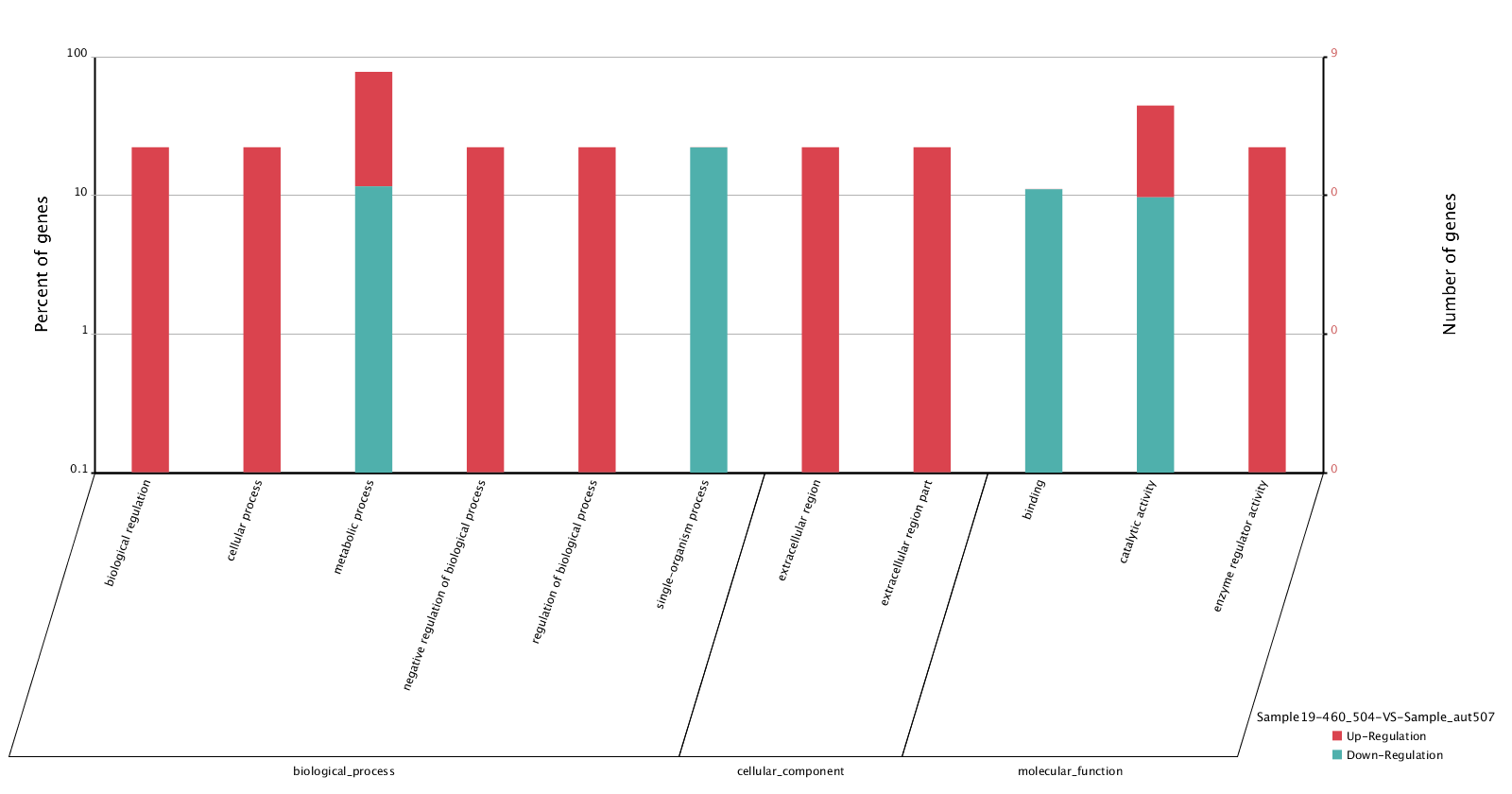

Supplement: Supplementary file 1 [file ijms-19-03718-s001.zip › Figure and Table/Figure S1/Sample19-460_504-VS-Sample_aut507.goclass.png]

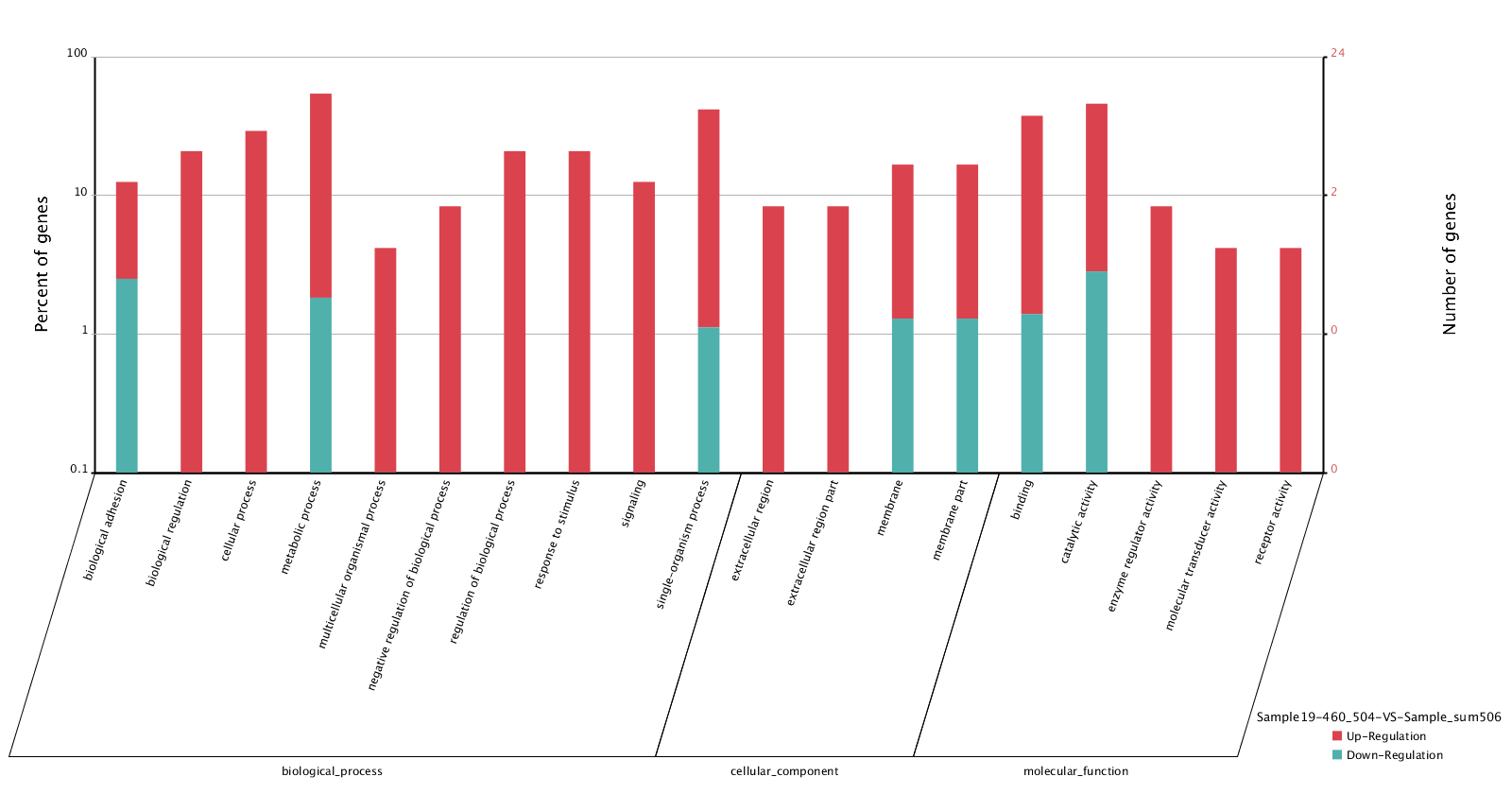

Supplement: Supplementary file 1 [file ijms-19-03718-s001.zip › Figure and Table/Figure S1/Sample19-460_504-VS-Sample_sum506.goclass.png]

# Top 20 Statistics of Pathway Enrichment for Sample10-710\_505-VS-Sample\_aut507

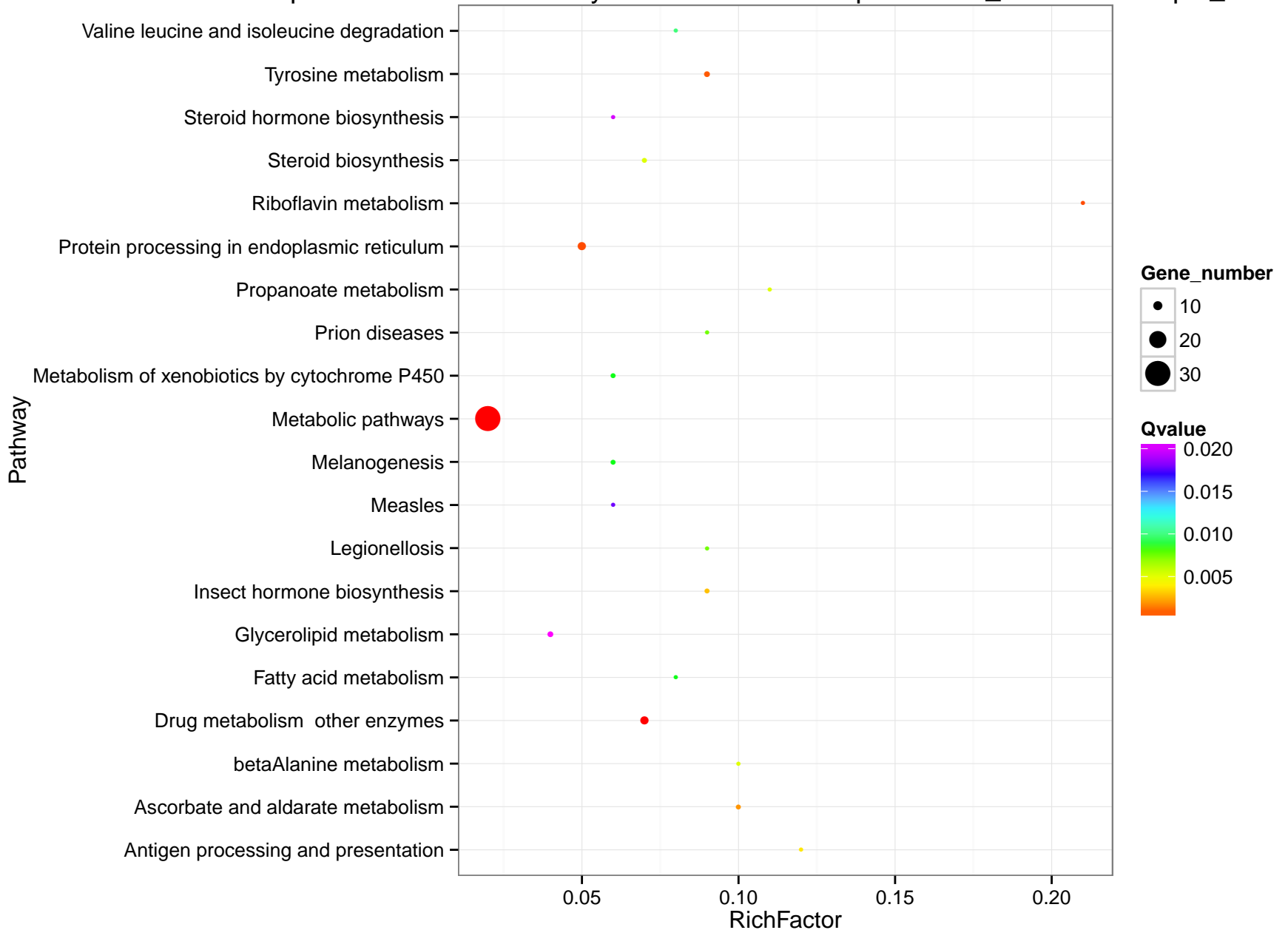

Supplement: Supplementary file 1 [file ijms-19-03718-s001.zip › Figure and Table/Figure S2/Sample10-710_505-VS-Sample_aut507.path.enrichment.pdf]

# Top 20 Statistics of Pathway Enrichment for Sample10-710\_505-VS-Sample\_sum506

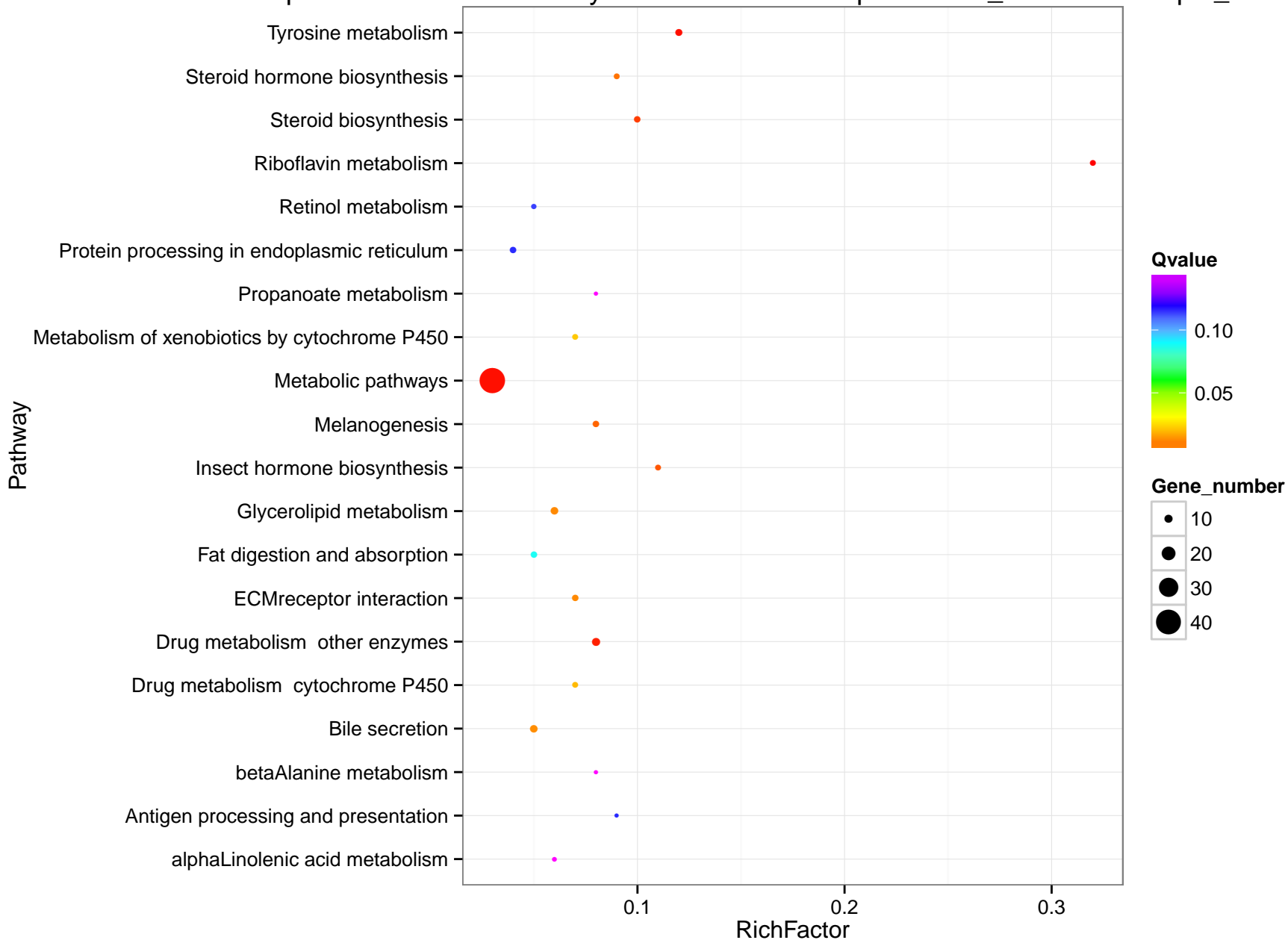

Supplement: Supplementary file 1 [file ijms-19-03718-s001.zip › Figure and Table/Figure S2/Sample10-710_505-VS-Sample_sum506.path.enrichment.pdf]

# Top 20 Statistics of Pathway Enrichment for Sample19-200\_506-VS-Sample872B\_50

Pathway

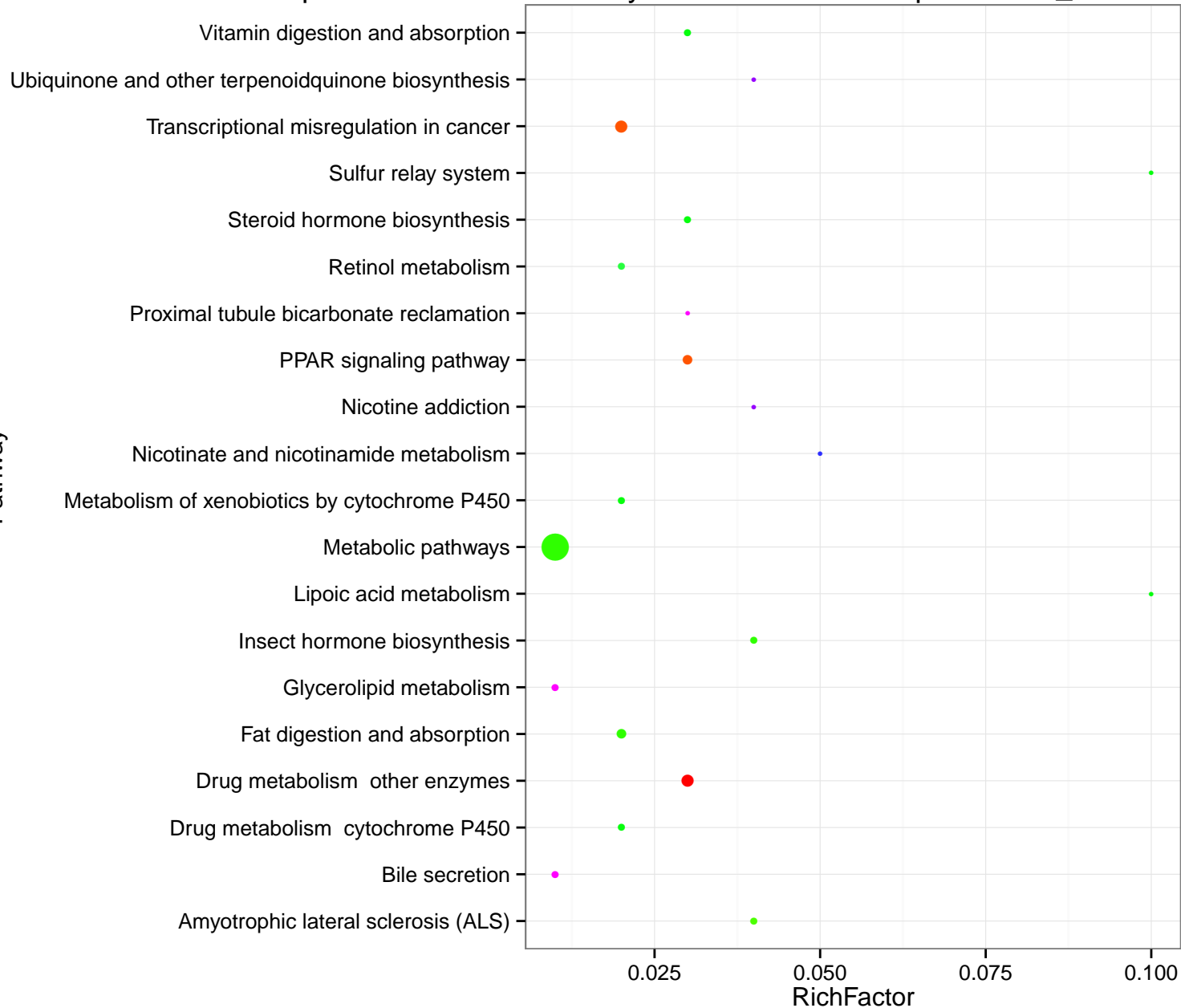

Gene\_number

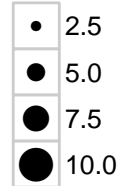

Qvalue

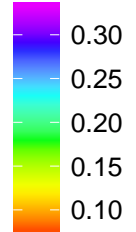

Supplement: Supplementary file 1 [file ijms-19-03718-s001.zip › Figure and Table/Figure S2/Sample19-200_506-VS-Sample872B_506.path.enrichment.pdf]

Top 20 Statistics of Pathway Enrichment for Sample19-200\_506-VS-Sample\_aut507

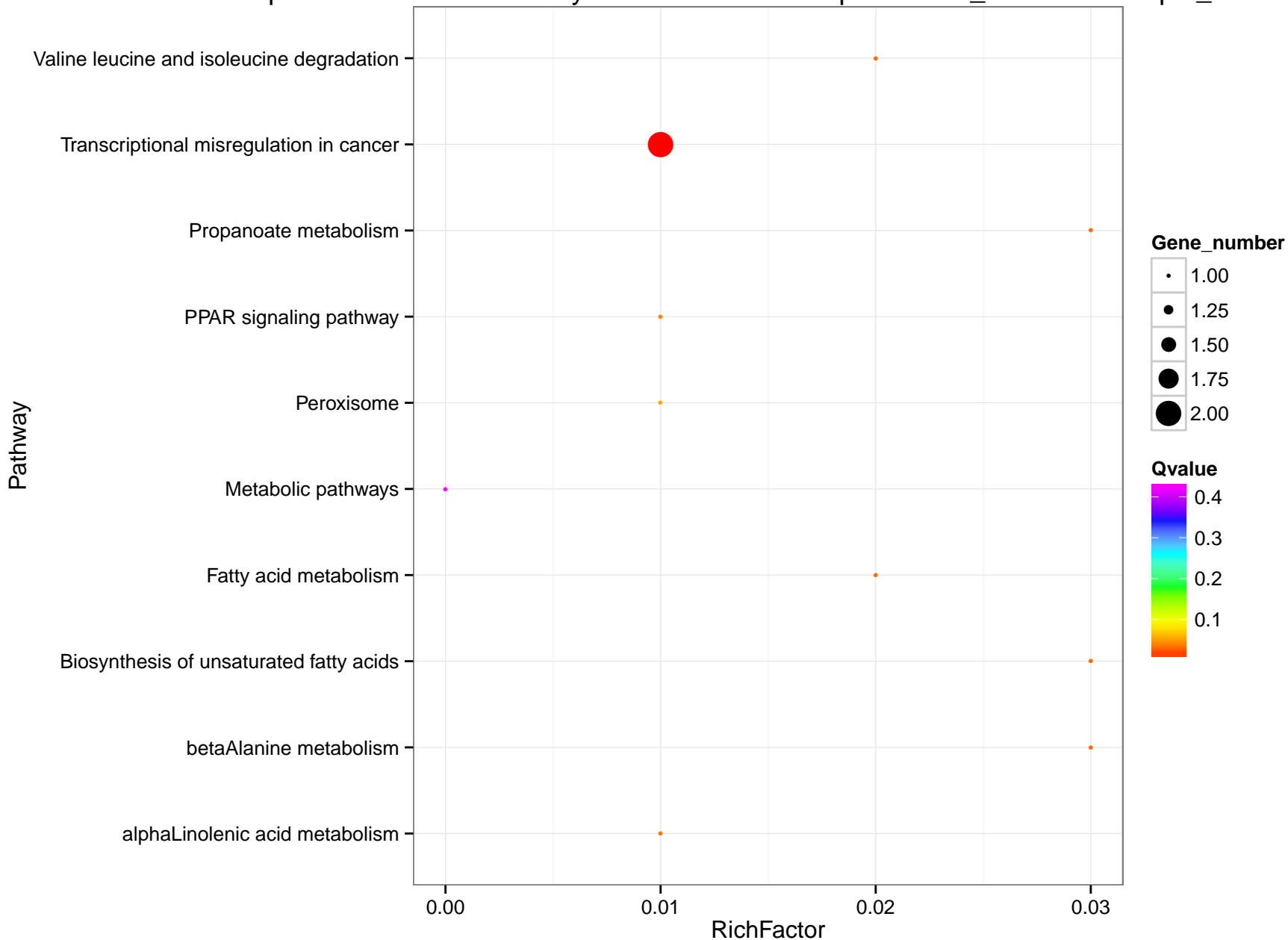

Supplement: Supplementary file 1 [file ijms-19-03718-s001.zip › Figure and Table/Figure S2/Sample19-200_506-VS-Sample_aut507.path.enrichment.pdf]

# Top 20 Statistics of Pathway Enrichment for Sample19-200\_506-VS-Sample\_sum506

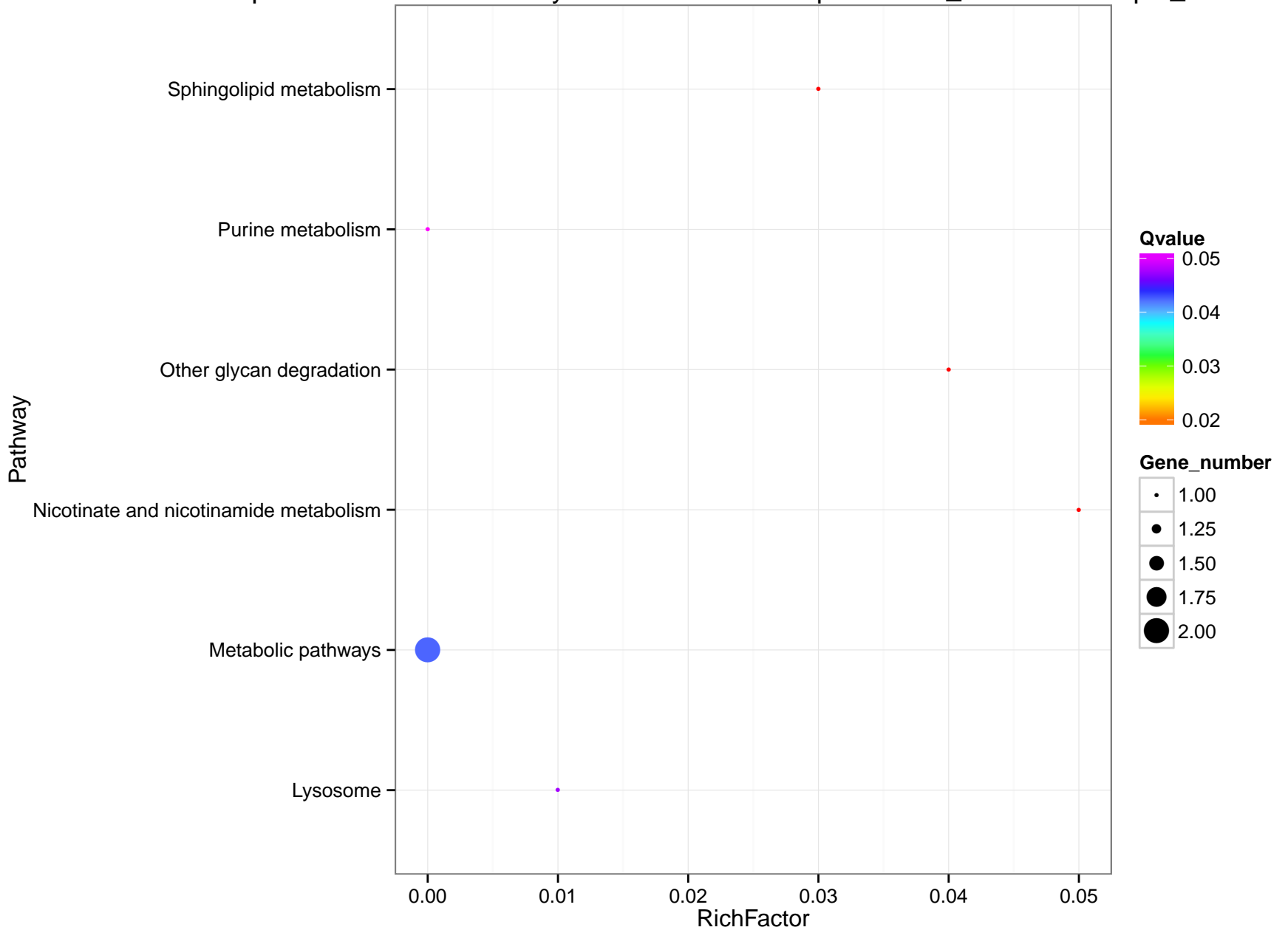

Supplement: Supplementary file 1 [file ijms-19-03718-s001.zip › Figure and Table/Figure S2/Sample19-200_506-VS-Sample_sum506.path.enrichment.pdf]

# Top 20 Statistics of Pathway Enrichment for Sample19-460\_504-VS-Sample872B\_506

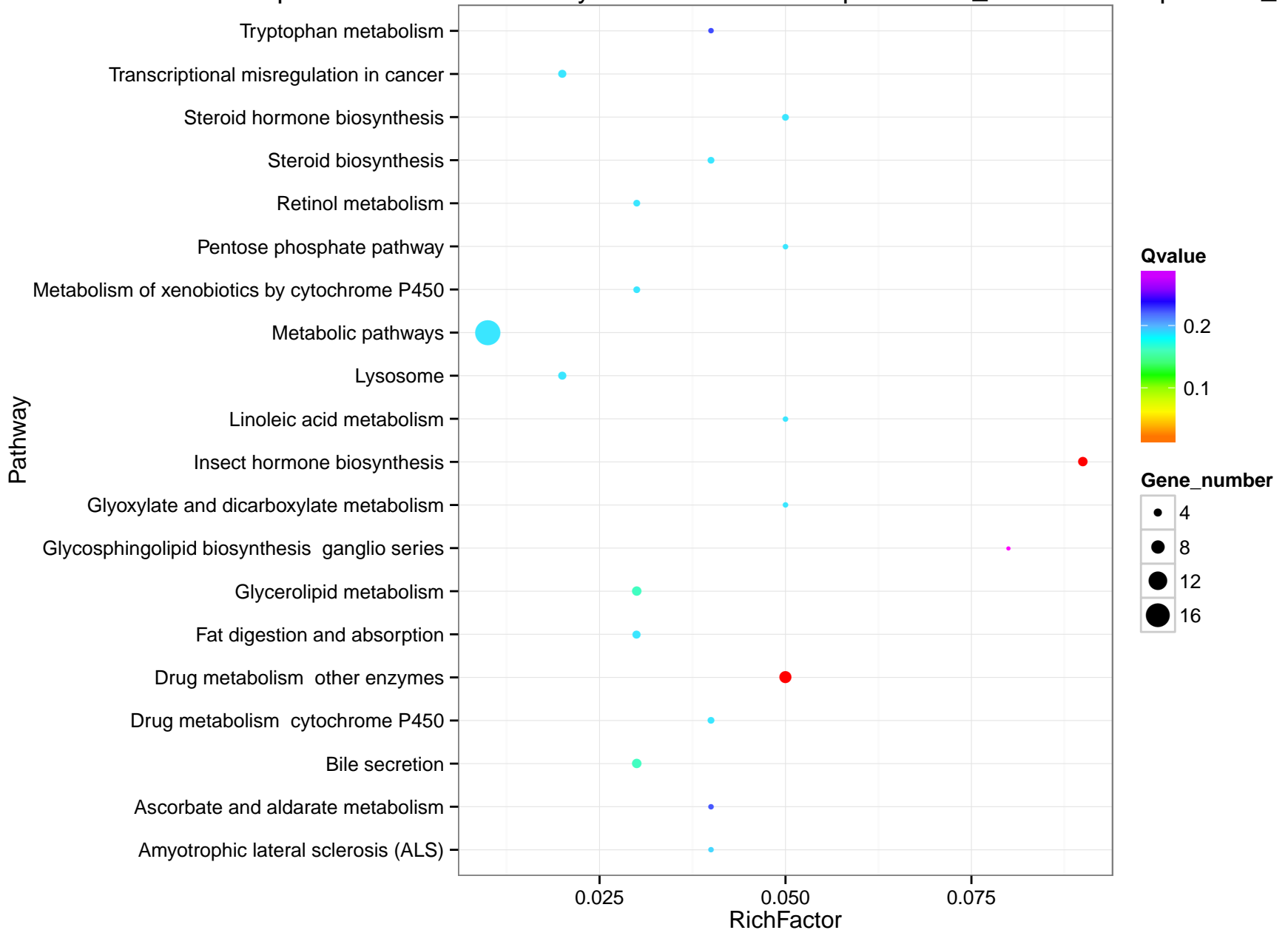

Supplement: Supplementary file 1 [file ijms-19-03718-s001.zip › Figure and Table/Figure S2/Sample19-460_504-VS-Sample872B_506.path.enrichment.pdf]

# Top 20 Statistics of Pathway Enrichment for Sample19-460\_504-VS-Sample\_aut507

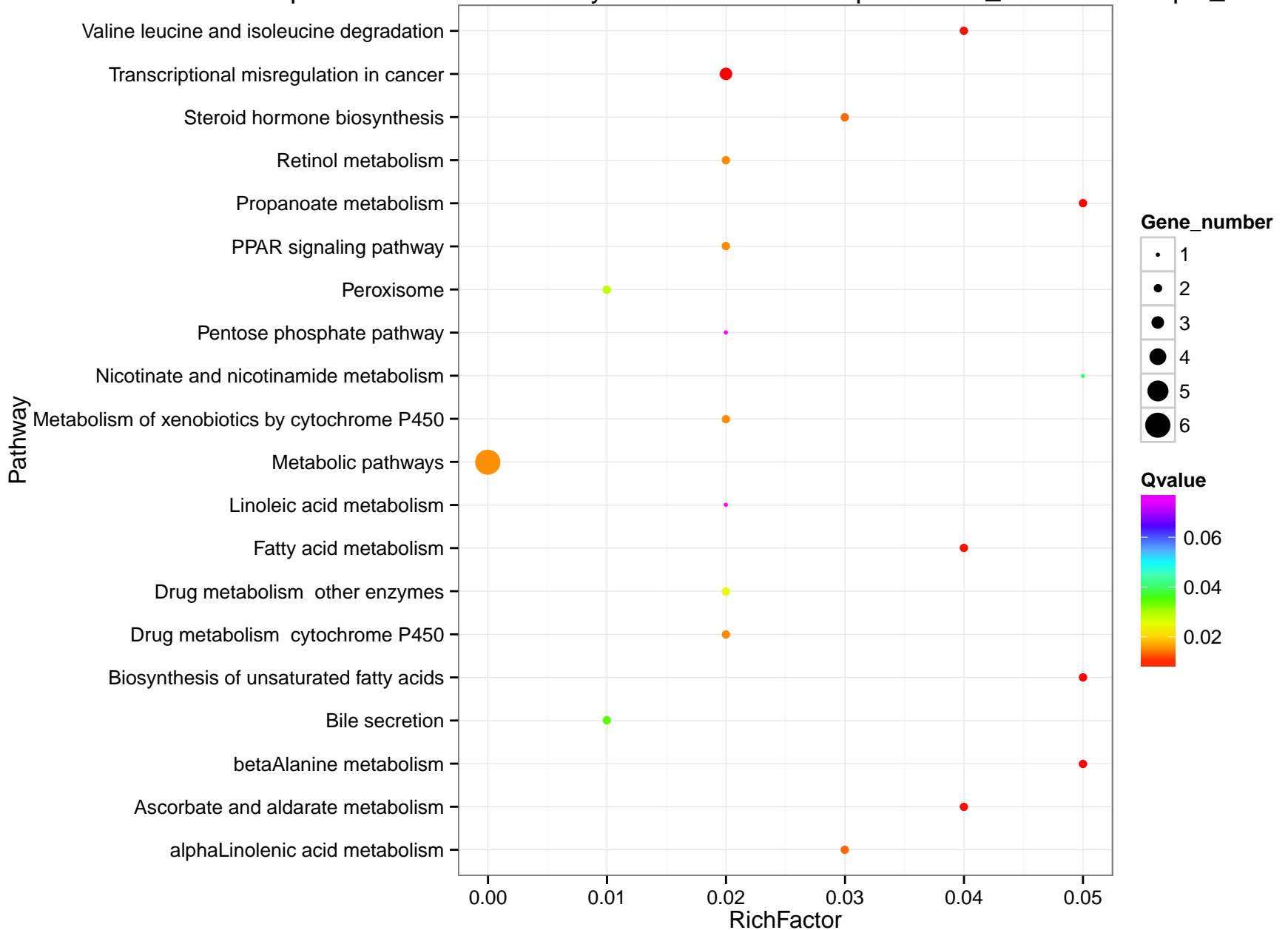

Supplement: Supplementary file 1 [file ijms-19-03718-s001.zip › Figure and Table/Figure S2/Sample19-460_504-VS-Sample_aut507.path.enrichment.pdf]

# Top 20 Statistics of Pathway Enrichment for Sample19-460\_504-VS-Sample\_sum506

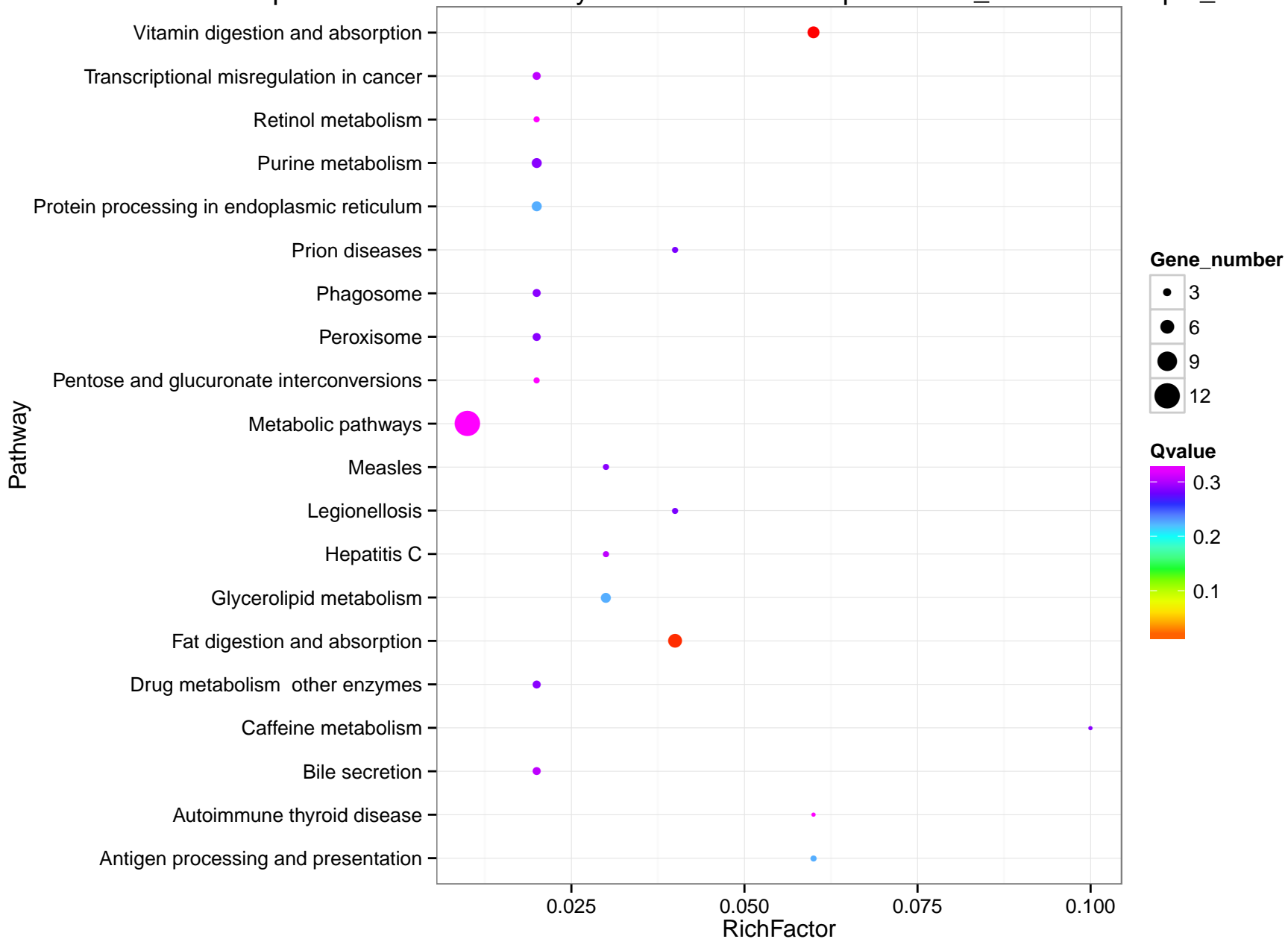

Supplement: Supplementary file 1 [file ijms-19-03718-s001.zip › Figure and Table/Figure S2/Sample19-460_504-VS-Sample_sum506.path.enrichment.pdf]

### BGIBMGA004399-MG

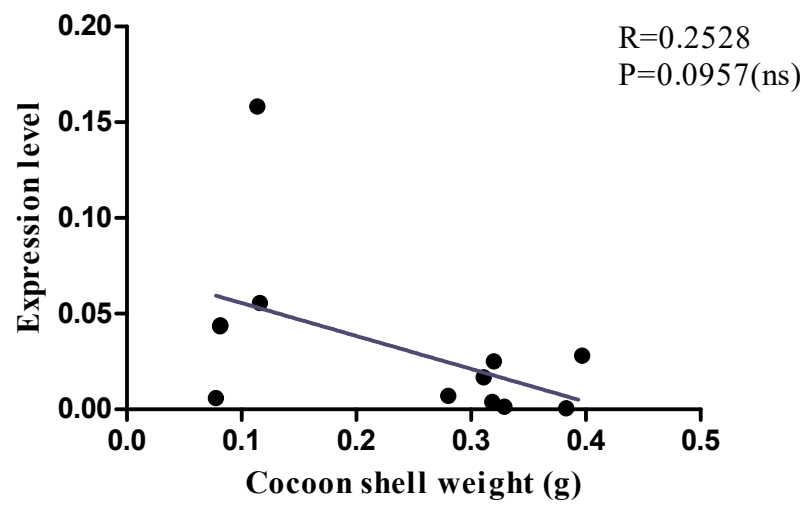

Supplement: Supplementary file 1 [file ijms-19-03718-s001.zip › Figure and Table/Figure S3/BGIBMGA004399-MG.pdf]

**BGIBMGA004399-PG**

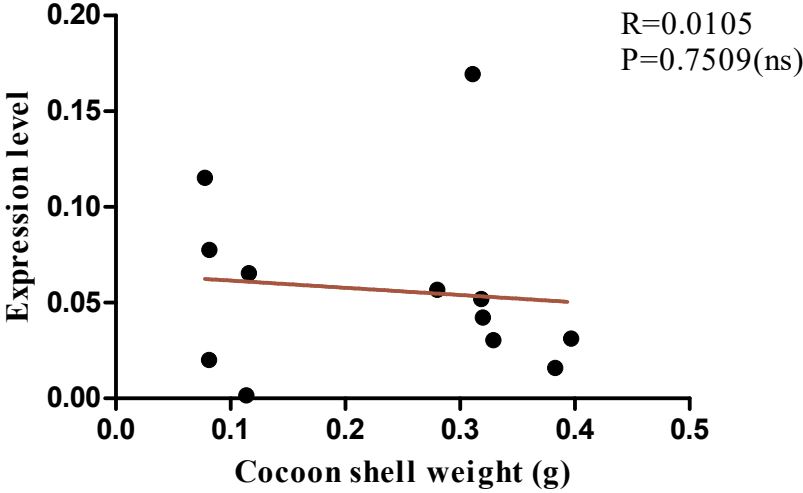

Supplement: Supplementary file 1 [file ijms-19-03718-s001.zip › Figure and Table/Figure S3/BGIBMGA004399-PG.pdf]

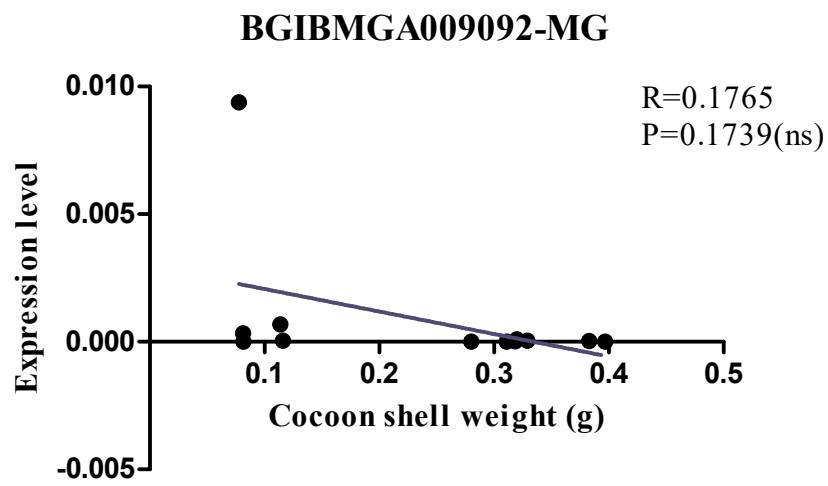

Supplement: Supplementary file 1 [file ijms-19-03718-s001.zip › Figure and Table/Figure S3/BGIBMGA009092-MG.pdf]

### BGIBMGA009092-PG

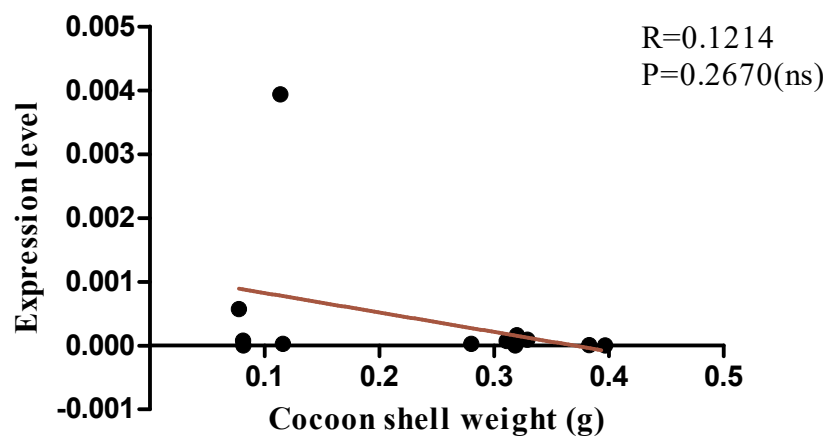

Supplement: Supplementary file 1 [file ijms-19-03718-s001.zip › Figure and Table/Figure S3/BGIBMGA009092-PG.pdf]

**BGIBMGA009093-MG**

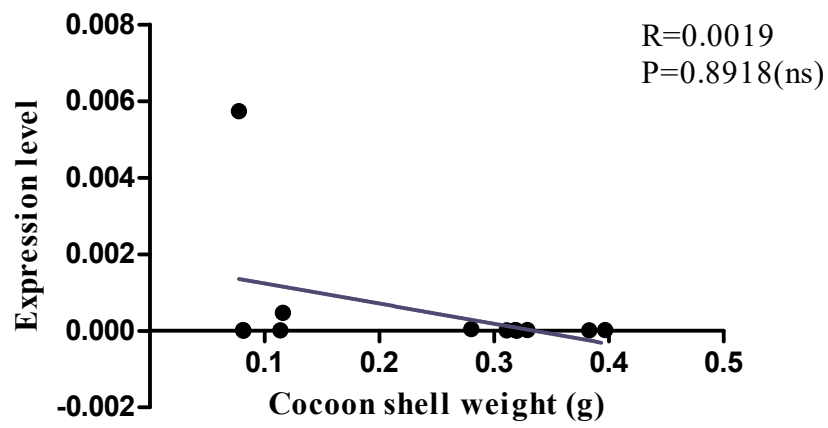

Supplement: Supplementary file 1 [file ijms-19-03718-s001.zip › Figure and Table/Figure S3/BGIBMGA009093-MG.pdf]

### BGIBMGA009093-PG

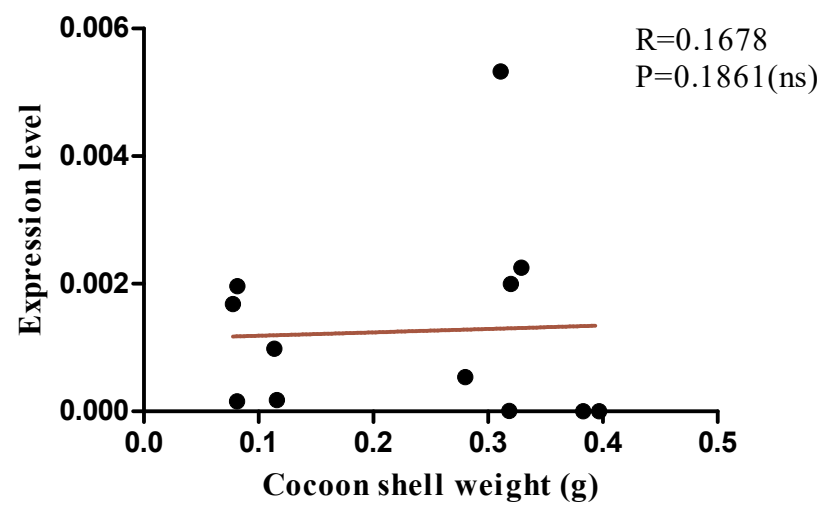

Supplement: Supplementary file 1 [file ijms-19-03718-s001.zip › Figure and Table/Figure S3/BGIBMGA009093-PG.pdf]
