# Supplementary material for: Identification of Genes that Control Silk Yield by RNA Sequencing Analysis of Silkworm (Bombyx mori) Strains of Variable Silk Yield
Source: Int J Mol Sci. 2018 Nov 22;19(12):3718. doi: 10.3390/ijms19123718 (PMC6321331; doi:10.3390/ijms19123718)
Supplement: Supplementary file 1 [file Sample10-710_505-VS-Sample872B_506.path.enrichment.pdf › Figure and Table/Figure S2/Sample10-710_505-VS-Sample872B_506.path.enrichment.pdf]

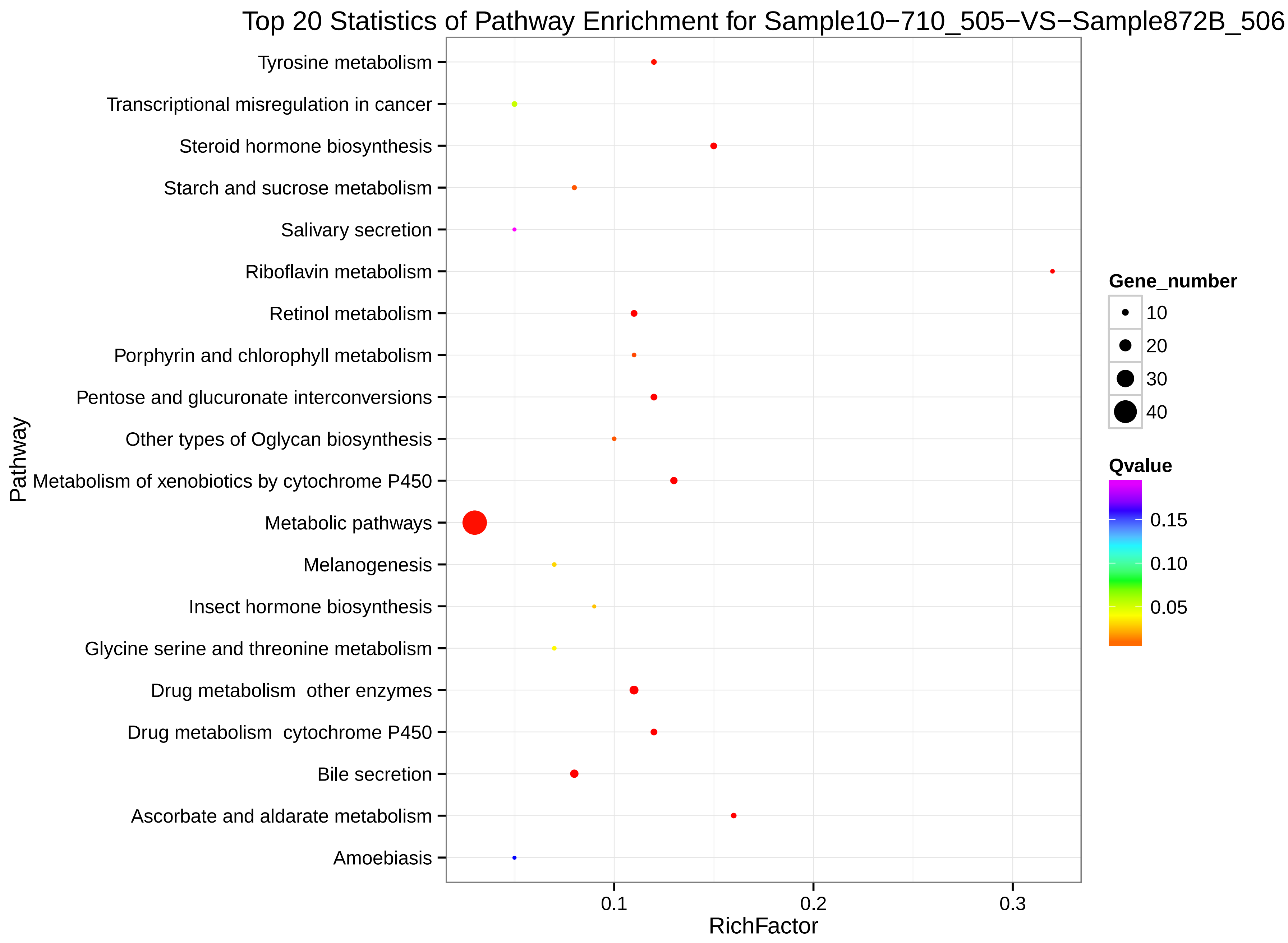
Top 20 Statistics of Pathway Enrichment for Sample10−710_505−VS−Sample872B_506
Pathway
Tyrosine metabolism
Transcriptional misregulation in cancer
Steroid hormone biosynthesis
Starch and sucrose metabolism
Salivary secretion
Riboflavin metabolism
Retinol metabolism
Porphyrin and chlorophyll metabolism
Pentose and glucuronate interconversions
Other types of Oglycan biosynthesis
Metabolism of xenobiotics by cytochrome P450
Metabolic pathways
Melanogenesis
Insect hormone biosynthesis
Glycine serine and threonine metabolism
Drug metabolism other enzymes
Drug metabolism cytochrome P450
Bile secretion
Ascorbate and aldarate metabolism
Amoebiasis
0.1
0.2
0.3
RichFactor
Gene_number
10
20
30
40
Qvalue
0.15
0.10
0.05
